# Power of Scanning Electron Microscopy and Energy Dispersive X-Ray Analysis in Rapid Microbial Detection and Identification at the Single Cell Level

**DOI:** 10.1038/s41598-020-59448-8

**Published:** 2020-02-11

**Authors:** Muhammad Saiful Islam Khan, Se-Wook Oh, Yun-Ji Kim

**Affiliations:** 1 Korea Food Research Institute, Consumer Safety Research Division, 55365, Wanju-Gun, Jeollabuk-Do Republic of Korea; 2 Department of Food and Nutrition, Kookmin University, 77 Jeongneung-ro, Seongbuk-gu, Seoul 02707 Republic of Korea; 3 Department of Food Biotechnology, University of Science and Technology, Daejeon, 305-350 Republic of Korea

**Keywords:** Pathogens, Natural hazards

## Abstract

The demand for rapid, consistent and easy-to-use techniques for detecting and identifying pathogens in various areas, such as clinical diagnosis, the pharmaceutical industry, environmental science and food inspection, is very important. In this study, the reference strains of six food-borne pathogens, namely, *Escherichia coli* 0157: H7 ATCC 43890, *Cronobacter sakazakii* ATCC 29004, *Salmonella* Typhimurium ATCC 43971, *Staphylococcus aureus* KCCM 40050, *Bacillus subtilis* ATCC 14579, and *Listeria monocytogenes* ATCC 19115, were chosen for scanning electron microscopy (SEM) and energy dispersive X-ray (EDX) analysis. In our study, the time-consuming sample preparation step for the microbial analysis under SEM was avoided, which makes this detection process notably rapid. Samples were loaded onto a 0.01-µm-thick silver (Ag) foil surface to avoid any charging effect. Two different excitation voltages, 10 kV and 5 kV, were used to determine the elemental information. Information obtained from SEM-EDX can distinguish individual single cells and detect viable and nonviable microorganisms. This work demonstrates that the combination of morphological and elemental information obtained from SEM-EDX analysis with the help of principal component analysis (PCA) enables the rapid identification of single microbial cells without following time-consuming microbiological cultivation methods.

## Introduction

Rapidly detecting and identifying biological threat microorganisms without traditional culture or chemical-based methods are highly important. The widely used identification techniques are nucleic acid-based, biosensor-based and immunologically based techniques. Real-time PCR multiplex PCR, loop-mediated isothermal amplification (LAMP), nucleic acid sequence-based amplification (NASBA), and oligonucleotide DNA microarray are examples of some common nucleic acid-based identification techniques^1–4^. These techniques have higher sensitivity, specificity, and reliability and can detect multiple pathogens in an automated manner with several constraints, such as sensitivity to PCR inhibitors and complicated primer design, and the methods cannot differentiate viable and nonviable cells^5,6^. All of these nucleic acid-based techniques are slow processes that require 4–72 h to detect microbes^1–6^. Electrochemical, optical and mass-based biosensors are commonly used to detect microbes. These automated, label-free, real-time detection processes can handle a large number of samples. Biosensor-based processes have several drawbacks, such as long incubation time, numerous washing steps, low specificity, interference with the food matrix and unsuitability for lesser cells^7–10^. The lateral flow immunoassay and enzyme-linked immunosorbent assay (ELISA) are two immunological-based detection techniques with several advantages and disadvantages; most importantly, these techniques are also slow processes that require 3–10 h^11,12^. These limitations increase the overall cost of the detection process due to costly logistics trails and restrict autonomous operation^13,14^. Some physical detection techniques, including basic fluorescence and laser-induced breakdown spectroscopy (LIBS), are prospective tools for differentiating biological from non-biological units but are not sufficiently capable for identification purposes^15–19^. In contrast, mass spectrometry-based techniques are capable of rapid detection and taxonomy of threat microorganisms^20–23^. Unfortunately, due to the exceedingly high sensitivity of mass-based detection, these methods cannot avoid false alarms^20,23^. Raman spectroscopy can address various drawbacks of traditional biological threat organism detection techniques with an ideal sensor to detect and identify multiple pathogens in real or near-real time^24^. To detect life-threatening pathogens, sensors must have sufficient sensitivity with low false alarm rates^24^. Raman analysis is very sensitive to bacterial growth conditions and has inadequate understanding of the taxonomic determination^24^. Both FTIR (Fourier transform infrared spectroscopy) and MALDI-TOF (matrix-assisted laser desorption/ionization time-of-flight) show potential for microbial identification. MALDI-TOF MS (mass spectrometry) is typically insensitive to variations in the procedure for the growing of microbes prior to the investigation and shows greater reliability in the identification results^25^. FTIR generates the fingerprint of the entire cell, and intraspecies variety may cause to overlying species boundaries that make the identification process complicated^26^. In contrast, in combination with this higher sensitivity, suitable bioinformatic processes enable identification below the species level, but strain-specific data of MALDI-TOF MS spectra could not be investigated with the identical processes applied for types identification^25^.

In recent studies, the application of electron microscopy (EM) is rare in the area of microbial detection, whereas EM played some crucial role in detecting the cause of infectious diseases in earlier studies^27^. EM is still an essential method that can help to detect and identify microorganisms with improved technology^28^. The major drawback of the use of electron microscopy was the method’s low sensitivity for various types of microbial research, especially the non-culturable specimens obtained from patients^29–31^. However, the recent development of filtration technique makes both scanning electron microscopy (SEM) and transmission electron microscopy (TEM) useful for the identification of pathogens. EM is a powerful analytical technique, even for identifying emerging pathogens, where there is no a priori information of the category of pathogens present^31^.

In the past, the difficulty with specimen preparation methods for SEM analysis was the major cause of its limited use in the routine analysis of microbiology^32,33^. Two other problems were also counted for achieving high-resolution SEM images of microbes: nonconducting behavior and the presence of moisture on microbial samples. Both of these reasons decrease the performance of the microscope, which reduces contrast and resolution^32,33^. Due to the drying problem, specimens become collapsed, shrunken and distorted, even after chemical fixation. ESEM (environmental scanning electron microscopy), wet-SEM, or cryo-techniques have been used to solve the drying problem along with solvent drying, critical point drying and/or freezing techniques^32,34–39^. Currently, ESEM is the most widely used technique for the analysis of biological samples in wet conditions^37^. Energy dispersive X-ray (EDX) microanalysis is an elemental analysis technique related to EM based on representative X-ray generation that determines the types of elements present in the target analyte. The EDX microanalysis is applied in diverse biomedical areas by numerous researchers and clinicians^40^. However, most of the scientific community is not completely conscious of EDX’s promising applications. The EDX can be considered a valuable instrument in every research that necessitates elemental determination, either endogenous or exogenous, in the tissue, cell or other new types of analyte^40^. The EDX detector represents the characteristic X-ray spectrum of a particular element that is a histogram plot of the number of counts against X-ray energy^40,41^. Qualitative and quantitative analysis, i.e., identification of elements in the spectrum and the amounts of each element present in the sample, is usually attained with the manufacturer’s software^41^. Conventionally, EDX is a powerful technique enabling an elemental analysis of the surface of the samples; thus, this technology can be suitable for the analysis of microbial samples because the microbes are originally very thin.

This study sought to demonstrate the power of the SEM-EDX analysis method for the identification and clear distinction among individual cells of six commonly encountered pathogenic microbes. The morphological information obtained from SEM and the chemical composition determined by EDX techniques for the same individual cell has been elucidated, and PCA analysis was performed to distinguish these findings. In this study, the method used neither any coating substance nor any fixative reagent, thereby making the analysis technique simple and rapid. To the best of the authors’ knowledge, this report describes the first application of this approach to characterize and distinguish pathogens at the single cell level.

## Results and Discussion

### SEM analysis of six food-borne pathogens

**Figure 1:**
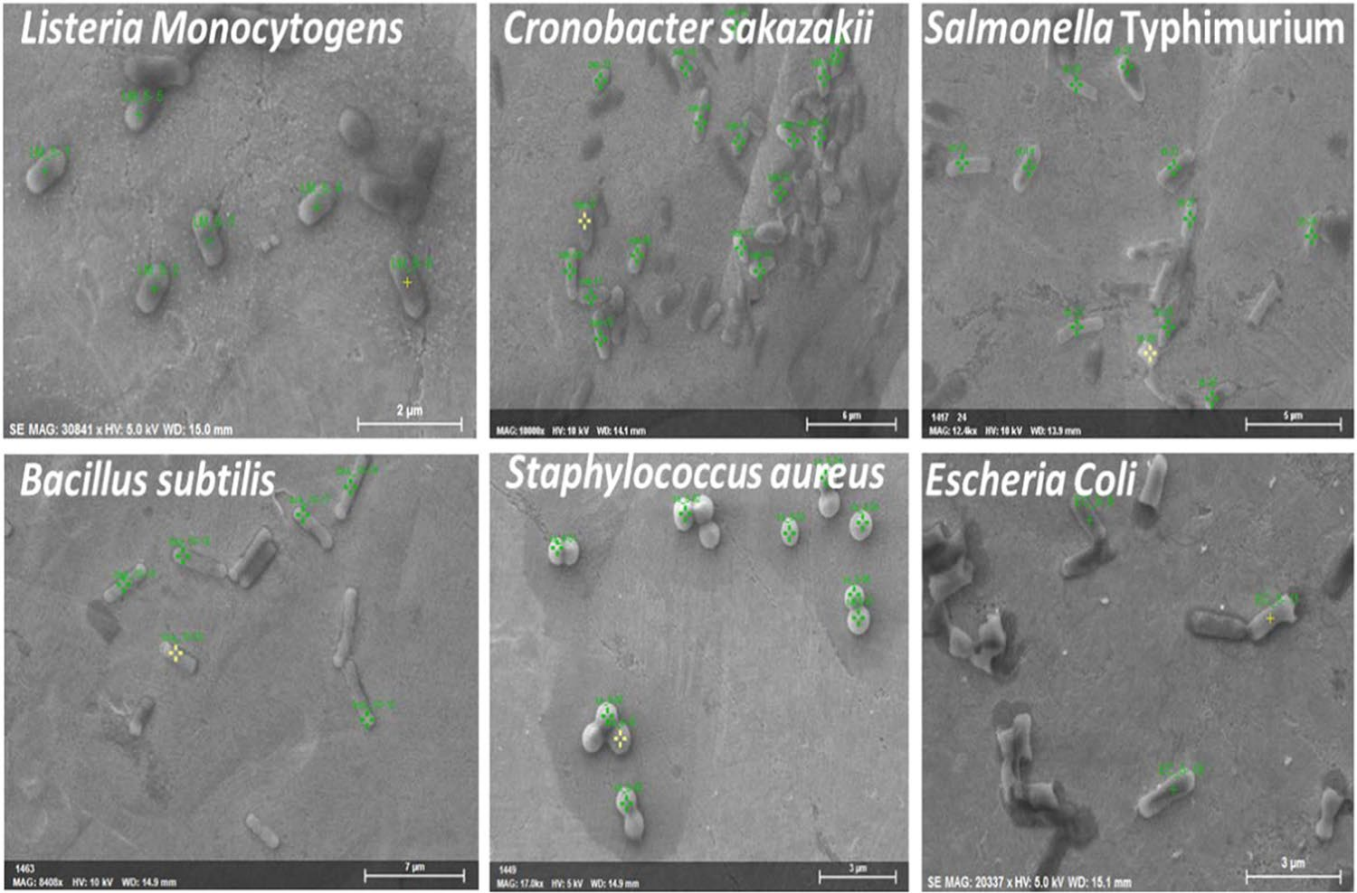
Secondary electron images (SEI) from SEM for six different types of foodborne pathogens. Samples were loaded on Ag foil, and no conductive coating was made.

A quantitative single-cell electron microscopic analysis, SEM-EDX, was employed to characterize six different types of reference pathogens. On average, 29 single cells of each reference sample, ranging from 20 to 36, were examined. As this study describes the first application of the combined technique of SEM-EDX for food-borne pathogen characterization, the unambiguous identification of pathogen types will be our major focus. Figure 1 shows the secondary electron images (SEIs) of the pathogen analyzed in this study. The morphologies obtained from SEIs were analyzed carefully; *Staphylococcus aureus* shows highly distinct morphology (round shape) among others. Hence, this microbe can be distinguished from the other five different types of pathogens analyzed in this study without further findings from EDX. SEIs are capable of distinguishing *Listeria monocytogens* and *Bacillus subtilis* using the data of their distinct size and shape compared to other three types, if the samples are previously known. The average size of *Listeria monocytogens* is 1.05 ± 0.98 µm and almost rectangular in shape, whereas the average size of *Bacillus subtilis* is 3.59 ± 0.96 µm and rod-like in shape (Table 1). Adequate amounts of expertise are not required for accurate prediction if *Listeria monocytogens* and *Bacillus subtilis* are present in the unknown sample. Table 1 shows that the measured sizes for *Escherichia coli*, *Salmonella* Typhimurium and *Cronobacter sakazakii* are somewhat similar. The similar sizes and rod-like shapes of these three types of pathogens make those difficult to distinguish among them; hence, only SEIs are not capable of distinguishing among these pathogens.

**Table 1:** Size of five different bacterial samples analyzed in this study.

Types of bacteria	Size Range (µm)	Avrg ± SD
*Bacillus subtilis* ATCC 14579	3.28–4.01	3.59 ± 0.96^a^
*Listeria monocytogenes* ATCC 19115	0.98–1.25	1.05 ± 0.98^b^
*Escherichia coli* 0157: H7 ATCC 43890	2.04–2.49	2.29 ± 0.98^c^
*Cronobacter sakazakii* ATCC 29004	1.95–2.36	2.13 ± 0.98^c^
*Salmonella* Typhimurium ATCC 43971	2.09–2.68	2.45 ± 0.98^c^

The size was measured from SEI. Average and standard deviations with different letters (a, b, c) in the same line were significantly different based on Duncan’s multiple range tests (*p* < 0.05).

### EDX analysis for five food-borne pathogens

**Figure 2:**
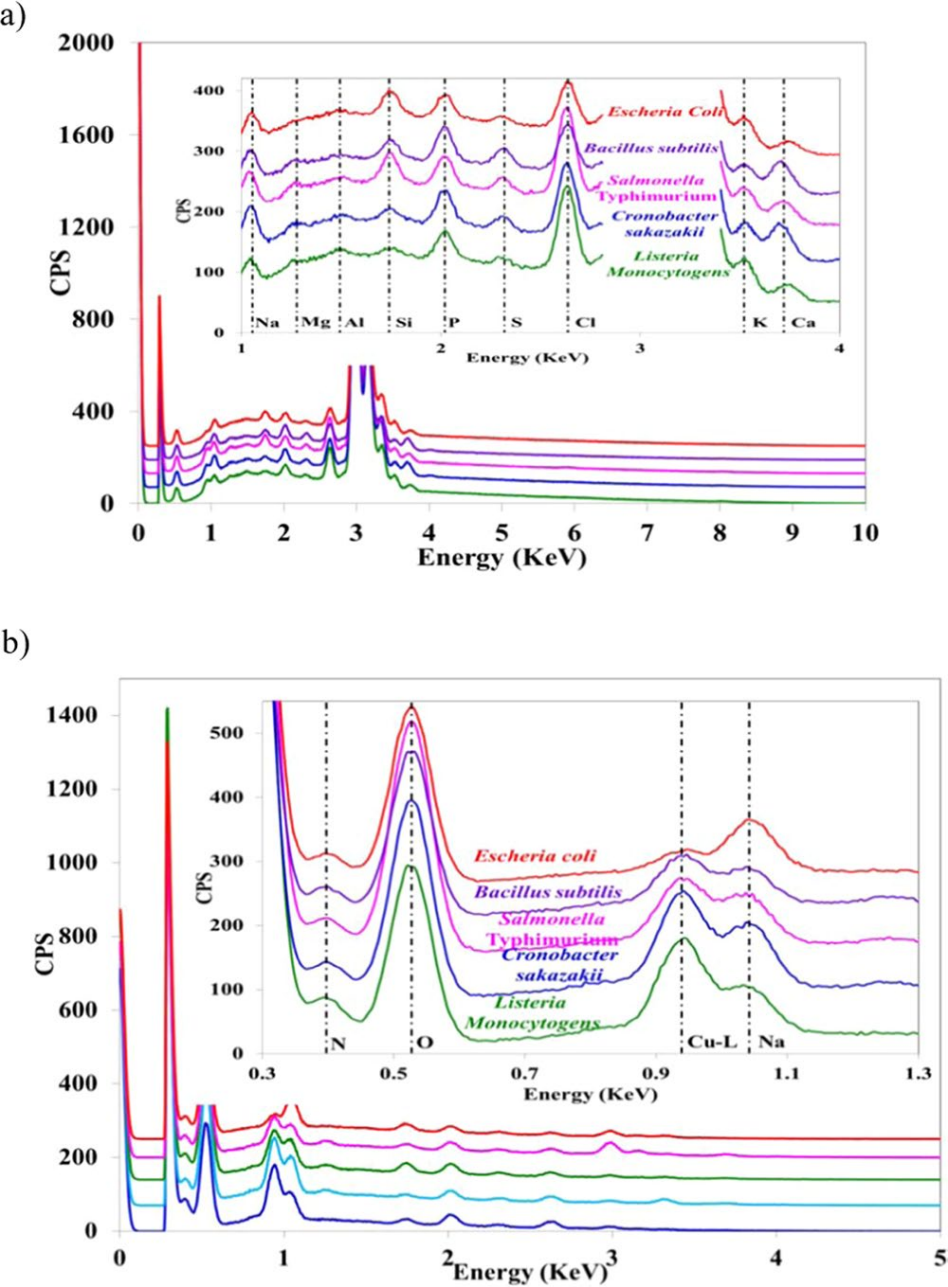
Typical X-ray spectrum of five individual pathogens of *Escherichia coli* 0157: H7 ATCC 43890, *Bacillus subtilis* ATCC 14579, *Salmonella* Typhimurium ATCC 43971, *Cronobacter sakazakii* ATCC 29004, and *Listeria monocytogenes* ATCC 19115 (single cell for each) obtained from EDX data. Magnified spectrum for the most significant area of each bacterium type shown as an inset, data for Ag-L line ~2.8 KeV to ~3.5 KeV were deleted for clarity. (**a**) EDX analysis performed at an accelerating voltage of 10 KeV and (**b**) EDX analysis performed at an accelerating voltage of 5 KeV.

Since energy dispersive X-ray (EDX) analysis is capable of detecting multiple elements and their weight percentages simultaneously, this technology was applied to detect five different types of individual bacterial cells analyzed in this study. Figure 2a,b shows the X-ray spectra of individual bacteria analyzed in this study (except *Staphylococcus aureus*) in point mode. A background study was performed to determine whether any differences were present in the point and area mode of the analysis (data not shown here) of the same pathogenic type. No differences were observed in the elemental information (either element type or the concentration of the elements present) in the case of point mode and area mode. Moreover, area mode requires more time to extract the elemental information, whereas point mode is notably rapid; within 30 s, the entire information can be extracted from the sample analyzed. Hence, in this study, the analysis was carried out in point mode, and throughout the manuscript, the analysis will refer point mode only if not mentioned otherwise. Each bacterium type has its own characteristic elemental composition, which is a key to distinguishing among pathogens (Fig. 2a,b). EDX provides elemental data in both atomic and weight percentages automatically through built-in software. In Fig. 2, we observed substantial amounts of silver (Ag) and copper (Cu) peak with other elements. The amount of Ag and Cu originated from the substrate, where the pathogen samples were loaded for SEM-EDX analysis. The final weight fractions values of different elements were normalized after deducting the weight fractions of silver (Ag) and copper (Cu) from the respective cells. Two different excitation energies, 10 kV and 5 kV, were used to obtain the entire elemental information of pathogens analyzed in this study. Using 10 kV did not acquire data on the nitrogen content present in the bacterial samples, whereas the lower energy of 5 kV was capable of showing the presence and amount of nitrogen in individual samples. The obtained weight fractions of the elements with higher atomic numbers, such as S, P, Cl, K, and Ca, decrease significantly with 5 kV^42^. Therefore, variation of the accelerating voltage is advantageous for distinguishing pathogenic microbes. According to the weight fractions of the elements C, N and O can be considered to be major elements present in all bacterial samples, the sum of these three elements would be in a range of 80 to 90 percent (Fig. 2). The rest of the elements can be considered to be minor elements, and the sum of their weight fractions would be in the range of 10 to 20 percent (Fig. 2). Quantitative amounts of the major and minor elements present in the bacterial sample play a major role in distinguishing among microbes. For instance, the amount of C present in *E. coli* is approximately 20%, whereas four other bacterial samples contained approximately 6–9% C when 10 kV was applied for data acquisition. Therefore, in the category of major components (C, N, and O), only *E. coli* can be distinguished among others at 10 kV. In the case of changing excitation energy, such as 5 kV, no bacterial samples showed any significant differences in the contents of major elements, such as C, N and O. In the category of minor elements, the presence of Na, Mg, Si, P, S, Cl, K, Ca and Mn were observed. At 10 kV, the K-content that is obtained (approximately 2.5%) in *Salmonella* Typhimurium is higher than any other types of bacterial samples analyzed, and the K-content could be the key for its distinction among the other four different types of pathogens. The amount of Ca can determine the presence of *Cronobacter sakazakii* with the combination of morphological information. *Cronobacter sakazakii* and *Bacillus subtilis* contain approximately 3% Ca, but *Bacillus subtilis* has a distinct size compared to the others. The presence of negligible amounts of Cl can be helpful in distinguishing *E. coli* from others in the category of minor elements. At 5 kV, Cl content can be used to distinguish *Listeria monocytogens* and *Salmonella* Typhimurium. Using the elemental ratio can be another useful means of distinguishing pathogens, such as approximately half of the amounts of Mg present in *E. coli* compared to the amount of Mg present in *Cronobacter sakazakii (*Fig. 2a). However, with the combined data regarding size, shape and elements present in the bacterial molecule, it is highly possible to identify and distinguish five pathogenic samples analyzed in this study, regardless of any prior expertise.

### PCA analysis of EDX data

**Figure 3:**
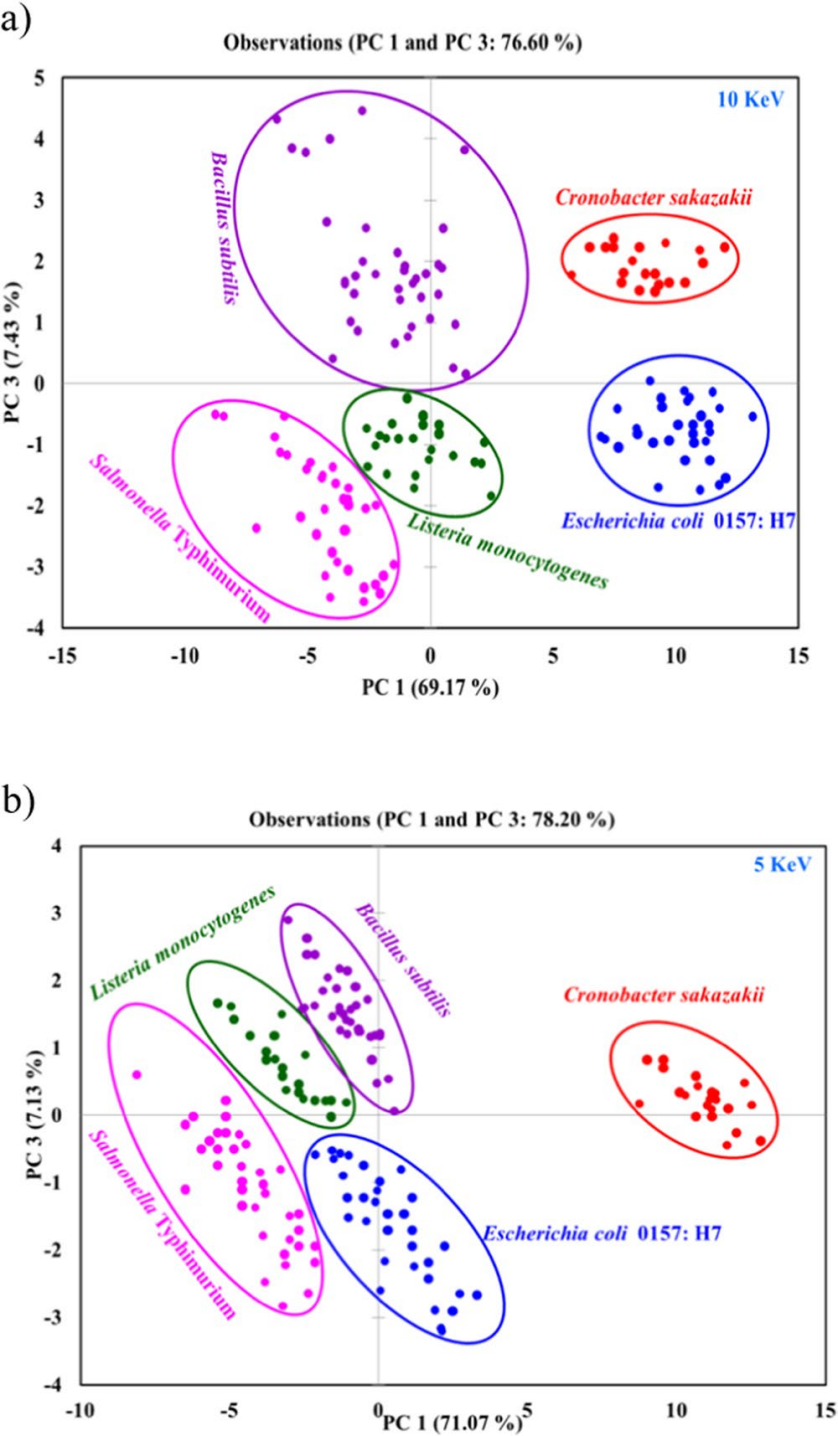
PC scatter plot of five different pathogen samples (single cell level) for PC1 and PC3, data obtained from EDX analysis. (**a**) EDX analysis performed at an accelerating voltage of 10 KeV, and (**b**) EDX analysis performed at an accelerating voltage of 5 KeV.

To identify and distinguish pathogens clearly, a multivariate modeling and analysis technique, principal component analysis (PCA), was performed for the EDX data using the XLSTAT program. Colored cluster PC scatter plots of EDX data generated from comparison of the two principal components (PCs) are depicted in Fig. 3a,b. EDX data for the five different types, such as *E. coli*, *Listeria monocytogens*, *Salmonella* Typhimurium, *Bacillus subtilis*, and *Cronobacter sakazakii*, of individual pathogens were plotted. The scatter plot of the PC1 and PC3 comparison revealed five distinct groups of samples with five different colors. Five different colors, such as blue, red, pink, green and purple, are significant for distinguishing *E. coli*, *Cronobacter sakazakii, Salmonella* Typhimurium, *Listeria monocytogens*, and *Bacillus subtilis*, respectively, for both 10 kV (Fig. 3a) and 5 kV (Fig. 3b) excitation energies. Few dots for *Listeria monocytogens* and *Salmonella* Typhimurium are difficult to distinguish, but the morphology findings will help to distinguish them if any ambiguity arises. *Listeria monocytogens* and *Bacillus subtilis* also share a narrow boundary, where ambiguity may arise for few cells to distinguish between those two, but morphology information in combination with EDX data can resolve any uncertainty. In Fig. 3b, the data ambiguity is greater compared to Fig. 3b; therefore, we can conclude that using 10 kV instead of 5 kV is advantageous for distinguishing among the bacterial samples. Using 5 kV could be advantageous if the focus is to measure the amounts of major elements, especially nitrogen^43,44^. From the above discussion, it can be stated that the PCA scatter plot obtained from EDX data for individual pathogens could be useful for its ability to distinguish among microbes unambiguously. Hence, it is certain that morphology in combination with elemental information has strong potential for unambiguously distinguishing among six types of food-borne pathogens analyzed in this study.

### Identification of individual microbes from a mixture

**Figure 4:**
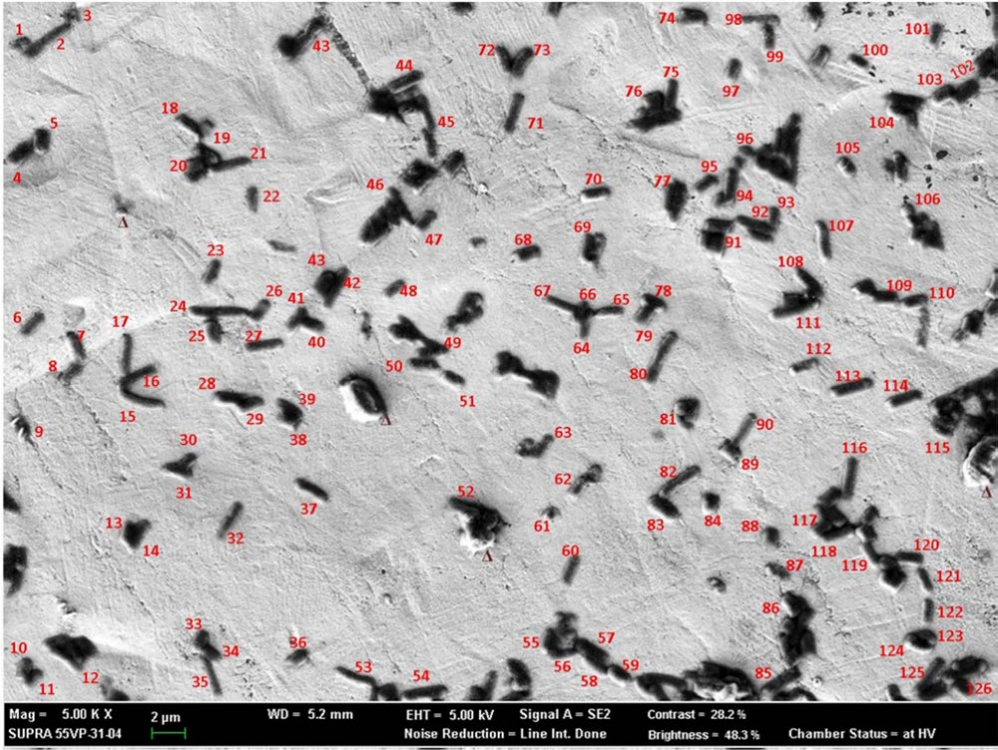
Secondary electron image (SEI) from SEM for the mixture of six different types of food-borne pathogens. Samples were loaded on Ag foil, and no conductive coating was made.

**Figure 5:**
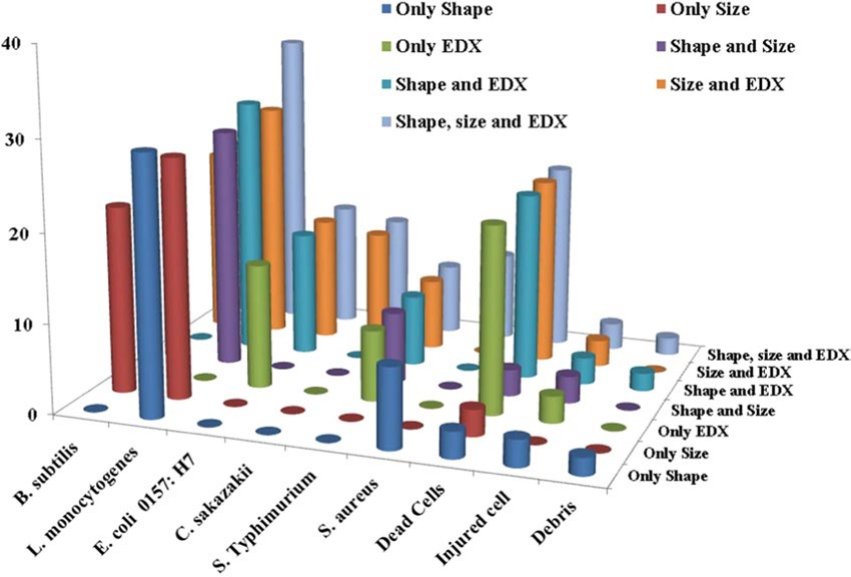
3D Number distribution plot of identified pathogens from the mixture of six different pathogens analyzed in this study. The number of identified bacterial types is plotted against the data on which the identification was made.

In another part of this study, analysis was performed by mixing all six types of pathogens, namely, *Escherichia coli* 0157: H7 ATCC 43890, *Cronobacter sakazakii* ATCC 29004, *Salmonella* Typhimurium ATCC 43971, *Staphylococcus aureus* KCCM 40050, *Bacillus subtilis* ATCC 14579, and *Listeria monocytogenes* ATCC 19115. Figure 4 shows the SEM image of the mixed sample; 126 well-separated individual cells were chosen for analysis to avoid any unwanted X-rays from the surrounding cell, which may provide misleading information for elemental concentration. Figure 5 shows the number of fractions of identified bacteria present in the mixture and the data on which the identification was made. All 126 bacteria were identified and distinguished by the combined data of morphology and the amounts of elements present in the pathogens obtained from SEM-EDX. Detailed information for the identification of 126 individual pathogens is given in Table S1.

Figure 4 shows the SEI of the mixture of six different bacterial cells; 126 pathogens were identified using all of the information extracted from SEM-EDX without any doubt regarding their respective group. Among the 126 pathogen cells, 21 were identified as *Bacillus subtilis* ATCC 14579, 34 were identified as *Listeria monocytogenes* ATCC 19115, 14 were identified as *E. coli* 0157: H7 ATCC 43890, 13 were identified as *Cronobacter sakazakii* ATCC 29004, 8 were identified as *Salmonella* Typhimurium ATCC 43971, 10 were identified as *Staphylococcus aureus* KCCM 40050 and the 26 remaining cells were identified as dead cells, injured cells and cell debris. The identification of *Bacillus subtilis* ATCC 14579 and *Staphylococcus aureus* KCCM 40050 19115 solely depends on the morphological results obtained from SEM. The majority of *Listeria monocytogenes* ATCC 19115 were identified based on their morphological information, but few required the combined information of morphology and elemental concentrations (Table S1). The remaining three, *E. coli* 0157: H7 ATCC 43890, *Cronobacter sakazakii* ATCC 29004, and *Salmonella* Typhimurium ATCC 43971, required morphology and elemental information in combination to confirm their detection. A PCA scatter plot (Figure not shown) of PC1 and PC3 components detects *E. coli* 0157: H7 ATCC 43890, *Cronobacter sakazakii* ATCC 29004, and *Salmonella* Typhimurium ATCC 43971 clearly in three different groups. Although some cells from *Listeria monocytogenes* ATCC 19115 and *Salmonella* Typhimurium ATCC 43971 are difficult to distinguish from the PCA plot, the identification was confirmed from the distinct size of *Listeria monocytogenes* ATCC 19115. However, this interference phenomenon is discussed elsewhere in this article. In Fig. 5, 3 particles were noted as triangular shapes, and those particles were identified as Si-based inorganic substances (based on their morphology and EDX). EDX analysis is capable of determining the viable and dead cells; morphological studies cannot provide clear data regarding viable or dead cells. As an example, in Fig. 4, particle nos. # 8, # 25, # 52, # 60, # 63, # 79, # 85, # 112, # 124, # 126 resembled live cells, whereas EDX determined only the presence of C and O, which ensures the death state of those pathogens, although the morphology shows that they are uninjured bacterial cells. The morphology study is capable of showing the real-time division of bacterial cell particle no. # 32 (Fig. 4B) and most of the cells in Fig. S1.

### SEM-EDX measurement without using any fixative reagent and metallic coating

SEM is one of the best analytical procedures to visualize sample morphology. This technique has a wide range of applications in various industrial, commercial and research fields and is becoming popular in the area of biological sample analysis^36–38,40^. A biological sample, such as a microorganism, contains sufficient amounts of water and shows very little conductivity. Therefore, conventional SEM shows some constraints for the direct analysis of microorganisms in their natural state under high vacuum conditions. For SEM observation, placing the sample in a high vacuum state is necessary, whereas a biological sample evaporates its surface water quickly in this state^43,44^. Hence, to observe microorganisms under SEM, fixing, dehydrating, and critical point drying of sample steps should be followed before placing the sample into a high vacuum state^35^. The fixative and dehydrating solutions contain several chemicals (such as osmium tetroxide and hexamethyldisilazane (HMDS)) that must penetrate to the entire specimen and remain with the sample after treatment. Therefore, during EDX measurement, the elements present in those fixative and dehydrating agents may interfere with the EDX spectra of the reference pathogens. The interfered EDX spectra require additional steps to correct the elemental information with the blank experimental result that may increase the percentage of error with the original elements present in the respective bacterial sample analyzed, as well as processing time. In this study, the bacterial samples were analyzed without using any fixative and dehydrating reagent to decrease identification time and any unwanted interference from elements in EDX spectra. The samples were dry under ambient conditions inside the biosafety cabinet. We successfully observed undistorted SEM images in Figs. 1, 4 and S1.

Another problem with a biological sample for SEM analysis is its non-conducting behavior, which creates charging around the sample^40^. Charging decreases the quality of the SEM image and reduces the effectiveness of the accelerating voltages. To avoid this charging effect, various materials are being used; such as carbon, gold, and platinum, to coat nonconducting samples to be analyzed that interfere with the original elements present in bacterial samples. In the present study, the samples were analyzed without any conducting coatings. Instead of conducting coating, the samples were loaded onto a conducting material surface, such as Ag foil, to allow the accumulated electrons to pass through it and thus minimize the charging effect. Figures 1, 4 and S1 demonstrate that the bacterial sample analyzed in this study is free from any charging. In the EDX spectra, the Ag peak appeared significantly at the higher accelerating voltage of 10 kV compared to 5 kV, and the elemental concentrations of the bacterial samples were calculated after subtracting the amount of Ag, and the values were normalized. Acquiring the Ag peak in the EDX spectra ensures that both the excitation energies penetrate the entire bacterial cell and that the elemental information present in the individual single cell is extracted, which signifies that there was no chance of missing any elemental information in either case. To the best of the authors’ knowledge, this report describes the first characterization of bacterial samples using SEM-EDX without both using fixative reagent and conducting coatings. Using fixative reagent is highly tedious and time-consuming; hence, this result may facilitate the analysis of bacterial samples in a shorter period of time. Most importantly, EDX analysis can be performed without any interruption of additional elemental peaks. Another advantage of using SEM is reducing the time of calculating microbial concentrations in solution, which had not been attempted previously. The typical method of determining the concentrations of microorganisms in a sample is to dilute the sample, grow the microbes on agar plates under certain conditions for several hours and count the colonies. SEM can determine bacterial cell count within several minutes, as the sample can be serially diluted, a certain volume of sample can be loaded onto the SEM sample holder, and the cells can be counted under SEM. In modern SEM, this counting can be performed automatically within several seconds. The cell count can be calculated with respect to liters, grams or as required to determine the concentration of bacterial cells in CFU (colony forming units). Thus, the application of SEM can reduce the tedious and time-consuming traditional culture method and can save time, money and workload.

In the present study, it has been displayed that different food-borne pathogens can be clearly identified at the single cell level by the combination of morphological and elemental concentration data obtained from SEM-EDX. However, it is not enough to undoubtedly identify all pathogen types when morphological or elemental information is used alone. This identification method is notably rapid and avoids traditional fixative, drying and conductive coating steps with several other advantages that are mentioned elsewhere in this section. The combined approach of these two devices can be complementary in the categorization of distinct pathogens. Although 6 different types of standard reference pathogens were studied in this work, the morphological and elemental data for these 6 types are not enough to clearly categorize all of the diverse types of pathogens. Certainly, there will be diverse challenges to identify pathogens collected from food and/or environmental samples. Due to environmental stress pathogens may show some modification both in their morphology^45,46^ and elemental informations. Addressing this issue requires conducting this kind of research continuously for other types of standard reference pathogens as well as pathogens collected from food and/or environment and obtaining a standard quality of EDX spectra for the pathogens that we did not study for the current research. The SEM-EDX information could be stowed in a library within our research group; therefore, in forthcoming work, we will attempt to make a good archive of pathogens that could be the key for making this detection and identification technique more robust. Although EDX peak for minor elements are very weak and the detection limits are rather high, this shortcomings of EDX data may not effect in the identification process of pathogens. Because, every pathogen has its own signature elemental configuration, which helps them to create different islands. We believe that in time, developments of SE energy spectroscopy in detector hardware and software will empower SEM-EDX even rapid and automatic detection of microbes.

## Materials and Methods

### Bacterial samples

Six commonly encountered food-borne pathogens, namely, *Escherichia coli* 0157: H7 ATCC 43890, *Cronobacter sakazakii* ATCC 29004, *Salmonella* Typhimurium ATCC 43971, *Staphylococcus aureus* KCCM 40050, *Bacillus subtilis* ATCC 14579, and *Listeria monocytogenes* ATCC 19115, were purchased for identification and discrimination. One colony of each bacterium was grown-up on Plate Count Agar (PCA, BD, Sparks, MD, USA) at 37 °C for 24 h and was taken to inject in previously prepared TSB (tryptic soy broth) solution (BD). The TSB solution was allowed to culture for 15–16 h at 37 °C inside an incubator. The culture was harvested followed by washing once through centrifugation at 10,000 × *g* for 10 min. Due to centrifugation the bacteria were settled on the bottom of the vessel, to detach the cell pellet deionized water (DW, 20 °C) was blown several times and the cell pellet was re-suspended and adjusted to about 4–5 log CFU/ml in water. A mixture of 6 different pathogens was prepared and adjusted to about 4–5 log CFU/ml in deionized water (DW, 20 °C). The prepared samples were stored at 4 °C before analysis.

### SEM-EDX instrumentation

To obtain morphological and elemental compositional data, the experiments were performed using an Auriga field emission scanning electron microscope (FEG SEM). The Auriga FEG SEM is a completely digital 30 kV Hi Resolution FEG SEM connected with EDX and a range of backscattered (BS) and secondary detectors peculiar to the instrument. The X-ray spectra were stored through the latest from the Oxford offering: Oxford - Advanced AZtecEnergy package with an SDD 127 eV. Auriga has several advantages over conventional SEM, such as excellent low kV imaging capabilities, and it could be employed with secondary electron and secondary ion detection in combination. Auriga’s on-axis lower voltage back scatter detector (EsB-energy selective BS detector) enables the inspection of BS electrons that leave the surface at a vertical angle to the service. The net X-ray intensities of the elements present in individual pathogens were obtained automatically by the application of a Monte Carlo calculation combined with reverse successive approximations^42,47,48^. The quantification process providing accurate results within an acceptable relative deviation. The combined information obtained from SEM and EDX can provide numerical data on the chemical compositions and the pathogens can be categorized based on their chemical components.

### Sample Preparation for SEM-EDX analysis

Sample preparation was carried out in a class II biosafety cabinet unless otherwise stated. To load the sample for SEM measurement, a conducting material, Ag foil (Sigma Aldrich, 0.01 mm, 99.9% trace metals basis), was used. Ag foil was cut (1 cm × 1 cm) and stacked with an SEM sample holder with double-sided glued tape. Several 10-µL spots of 6 previously stored individual bacterial suspensions at 4–5 log CFU/ml and the mixture of 6 pathogens were poured onto the 7 different foils. The samples were left inside the biosafety cabinet for several minutes until the moisture was removed completely.

### SEM-EDX measurements

To attain ideal measurement conditions, i.e., low background level and high sensitivity, for the analysis of the elements with lower atomic numbers, such as C, N and O, accelerating voltages of 10 kV and 5 kV and 1.0 nA beam current were used. The working distance and lens aperture sizes were 8 mm and 30 μm, respectively. To acquire a statistically significant number of counts in the X-ray spectra, a distinctive quantifying time of 30 s for each point was used. During area (0.05 µm × 0.05 µm) mode analysis, the measurement was carried out for a minimum of 10 min.

### Principal Component Analysis (PCA)

Principal component analysis (PCA), using XLSTAT- Base solution program 2018, was performed on the chemical compositions obtained for each individual particle using low-*Z* particle EPMA to determine whether these quantitative results were sufficient to distinguish among the six individual pathogenic microbes^49,50^.

### Statistical analysis

All of the data acquired in this study were studied by the SAS program (SAS Institute Inc.). Every measurement had 3 repeats. If ANOVA showed significant treatment properties, Duncan’s multiple range test was performed to compare the averages at *p* < 0.05.

## Supplementary information

Supplementary Information.
