# Supplementary material for: Power of Scanning Electron Microscopy and Energy Dispersive X-Ray Analysis in Rapid Microbial Detection and Identification at the Single Cell Level
Source: Sci Rep. 2020 Feb 11;10:2368. doi: 10.1038/s41598-020-59448-8 (PMC7012924; doi:10.1038/s41598-020-59448-8)
Supplement: Supplementary file 1 — Supplementary Information. [file 41598_2020_59448_MOESM1_ESM.docx]

**Power of Scanning Electron Microscopy and Energy Dispersive X-Ray Analysis in Rapid Microbial Detection and Identification at the Single Cell Level**

Muhammad Saiful Islam Khan^1^, Se-Wook Oh^2^, Yun-Ji Kim^1,3,*^

^1^Korea Food Research Institute, Wanju-Gun, Jeollabuk-Do, Republic of Korea

^2^Department of Food and Nutrition, Kookmin University, 77 Jeongneung-ro, Seongbuk-gu, Seoul, 02707, Republic of Korea

^3^Department of Food Biotechnology, University of Science and Technology, Daejeon, 305-350, Republic of Korea

**Supplementary information**

Figure 1S: SEI of *Listeria monocytogenes* ATCC 19115. Most of the cells are in the stage of its division. Samples were loaded on Ag foil, and no conductive coating was made.


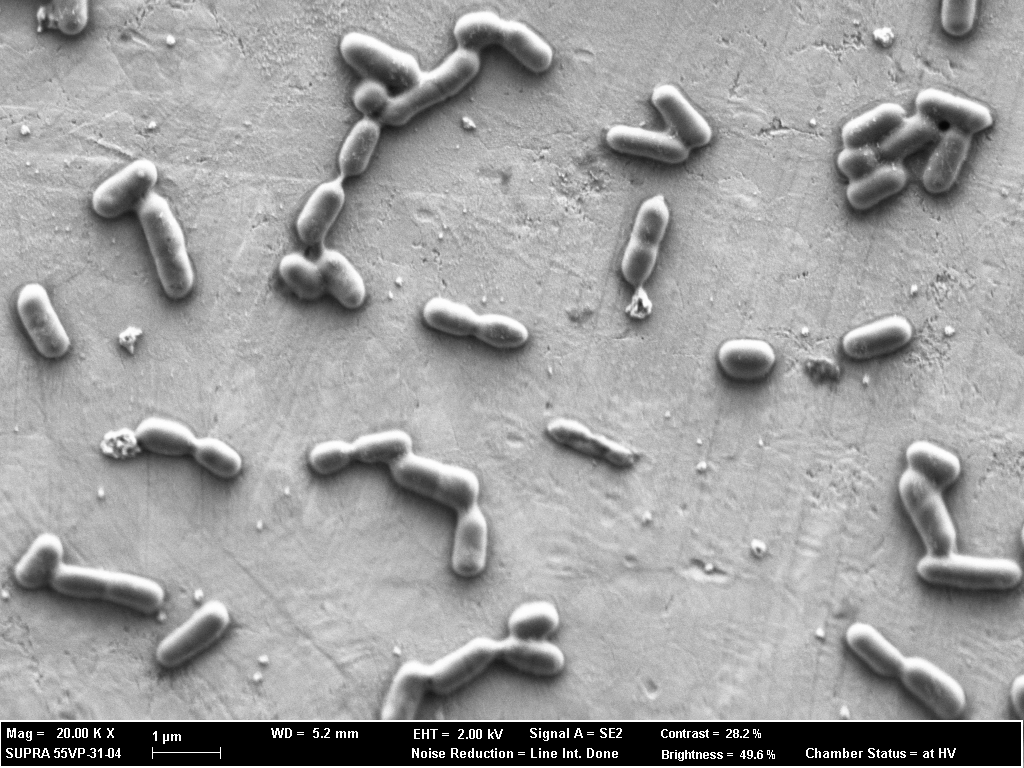


Table 1S: Pathogen identification from the mixture based on the data obtained from SEM-EDX.

| Particle no. | Inference from | | | | | | | |
| --- | --- | --- | --- | --- | --- | --- | --- | --- |
|  | Only Shape | Only Size | Only EDX | Shape and Size | Shape and EDX | Size and EDX | Shape, size and EDX | Identified Bacteria |
| 1 | ✓ | × | × | × | × | × | × | [*S.* *aureus*](https://www.google.co.kr/search?biw=1920&bih=934&q=staphylococcus+aureus&spell=1&sa=X&ved=0ahUKEwjTp_GboovPAhXEm5QKHYQJBgkQvwUIFygA) KCCM 40050 |
| 2 | × | ✓ | × | ✓ | × | ✓ | ✓ | [*B. subtilis*](https://www.google.co.kr/url?sa=t&rct=j&q=&esrc=s&source=web&cd=1&cad=rja&uact=8&ved=0ahUKEwihsbGnoovPAhVBGJQKHfQwDV8QFggdMAA&url=https%3A%2F%2Fen.wikipedia.org%2Fwiki%2FBacillus_subtilis&usg=AFQjCNGFijWm9LtsdZ-Da63OlFj9u3JOdg&sig2=w2ehAcPeNMdxfmDxDgXppA&bvm=bv.132653024,d.dGo)  ATCC 14579 |
| 3 | ✓ | × | × | × | × | × | × | [*S.* *aureus*](https://www.google.co.kr/search?biw=1920&bih=934&q=staphylococcus+aureus&spell=1&sa=X&ved=0ahUKEwjTp_GboovPAhXEm5QKHYQJBgkQvwUIFygA) KCCM 40050 |
| 4 | ✓ | ✓ | × | ✓ | ✓ | ✓ | ✓ | [*L. monocytogenes*](https://www.google.co.kr/url?sa=t&rct=j&q=&esrc=s&source=web&cd=1&cad=rja&uact=8&ved=0ahUKEwjr1om9oovPAhWCoJQKHQXfAHcQFggiMAA&url=https%3A%2F%2Fen.wikipedia.org%2Fwiki%2FListeria_monocytogenes&usg=AFQjCNGclwOGCJ_vA7pTFhCwISnMtBS3kg&sig2=U-NJNe3xcw6TWk9ePvp35g) ATCC 19115 |
| 5 | ✓ | ✓ | × | ✓ | ✓ | ✓ | ✓ | [*L. monocytogenes*](https://www.google.co.kr/url?sa=t&rct=j&q=&esrc=s&source=web&cd=1&cad=rja&uact=8&ved=0ahUKEwjr1om9oovPAhWCoJQKHQXfAHcQFggiMAA&url=https%3A%2F%2Fen.wikipedia.org%2Fwiki%2FListeria_monocytogenes&usg=AFQjCNGclwOGCJ_vA7pTFhCwISnMtBS3kg&sig2=U-NJNe3xcw6TWk9ePvp35g) ATCC 19115 |
| 6 | × | × | × | × | × | ✓ | ✓ | [*C. sakazakii*](https://www.google.co.kr/url?sa=t&rct=j&q=&esrc=s&source=web&cd=1&cad=rja&uact=8&ved=0ahUKEwjYuJDsoYvPAhXONpQKHUTkDh4QFggfMAA&url=https%3A%2F%2Fen.wikipedia.org%2Fwiki%2FCronobacter_sakazakii&usg=AFQjCNG4PxVztbOnpuuiy-BQnzqAysZdrw&sig2=jWhylTWYc9rD6Be3xz2CLQ)  ATCC 29004 |
| 7 | × | × | × | × | × | ✓ | ✓ | [*C. sakazakii*](https://www.google.co.kr/url?sa=t&rct=j&q=&esrc=s&source=web&cd=1&cad=rja&uact=8&ved=0ahUKEwjYuJDsoYvPAhXONpQKHUTkDh4QFggfMAA&url=https%3A%2F%2Fen.wikipedia.org%2Fwiki%2FCronobacter_sakazakii&usg=AFQjCNG4PxVztbOnpuuiy-BQnzqAysZdrw&sig2=jWhylTWYc9rD6Be3xz2CLQ)  ATCC 29004 |
| 8 | × | × | ✓ | × | ✓ | ✓ | ✓ | Dead Cell |
| 9 | ✓ | × | × | × | ✓ | × | ✓ | Debris |
| 10 | ✓ | ✓ | × | ✓ | ✓ | ✓ | ✓ | [*L. monocytogenes*](https://www.google.co.kr/url?sa=t&rct=j&q=&esrc=s&source=web&cd=1&cad=rja&uact=8&ved=0ahUKEwjr1om9oovPAhWCoJQKHQXfAHcQFggiMAA&url=https%3A%2F%2Fen.wikipedia.org%2Fwiki%2FListeria_monocytogenes&usg=AFQjCNGclwOGCJ_vA7pTFhCwISnMtBS3kg&sig2=U-NJNe3xcw6TWk9ePvp35g) ATCC 19115 |
| 11 | ✓ | ✓ | × | ✓ | ✓ | ✓ | ✓ | [*L. monocytogenes*](https://www.google.co.kr/url?sa=t&rct=j&q=&esrc=s&source=web&cd=1&cad=rja&uact=8&ved=0ahUKEwjr1om9oovPAhWCoJQKHQXfAHcQFggiMAA&url=https%3A%2F%2Fen.wikipedia.org%2Fwiki%2FListeria_monocytogenes&usg=AFQjCNGclwOGCJ_vA7pTFhCwISnMtBS3kg&sig2=U-NJNe3xcw6TWk9ePvp35g) ATCC 19115 |
| 12 | × | ✓ | × | ✓ | × | ✓ | ✓ | [*B. subtilis*](https://www.google.co.kr/url?sa=t&rct=j&q=&esrc=s&source=web&cd=1&cad=rja&uact=8&ved=0ahUKEwihsbGnoovPAhVBGJQKHfQwDV8QFggdMAA&url=https%3A%2F%2Fen.wikipedia.org%2Fwiki%2FBacillus_subtilis&usg=AFQjCNGFijWm9LtsdZ-Da63OlFj9u3JOdg&sig2=w2ehAcPeNMdxfmDxDgXppA&bvm=bv.132653024,d.dGo)  ATCC 14579 |
| 13 | × | × | ✓ | × | ✓ | ✓ | ✓ | Dead Cell |
| 14 | × | × | ✓ | × | ✓ | ✓ | ✓ | Dead cell |
| 15 | × | ✓ | × | ✓ | × | ✓ | ✓ | [*B. subtilis*](https://www.google.co.kr/url?sa=t&rct=j&q=&esrc=s&source=web&cd=1&cad=rja&uact=8&ved=0ahUKEwihsbGnoovPAhVBGJQKHfQwDV8QFggdMAA&url=https%3A%2F%2Fen.wikipedia.org%2Fwiki%2FBacillus_subtilis&usg=AFQjCNGFijWm9LtsdZ-Da63OlFj9u3JOdg&sig2=w2ehAcPeNMdxfmDxDgXppA&bvm=bv.132653024,d.dGo)  ATCC 14579 |
| 16 | × | ✓ | × | ✓ | × | ✓ | ✓ | [*B. subtilis*](https://www.google.co.kr/url?sa=t&rct=j&q=&esrc=s&source=web&cd=1&cad=rja&uact=8&ved=0ahUKEwihsbGnoovPAhVBGJQKHfQwDV8QFggdMAA&url=https%3A%2F%2Fen.wikipedia.org%2Fwiki%2FBacillus_subtilis&usg=AFQjCNGFijWm9LtsdZ-Da63OlFj9u3JOdg&sig2=w2ehAcPeNMdxfmDxDgXppA&bvm=bv.132653024,d.dGo)  ATCC 14579 |
| 17 | × | ✓ | × | ✓ | × | ✓ | ✓ | [*B. subtilis*](https://www.google.co.kr/url?sa=t&rct=j&q=&esrc=s&source=web&cd=1&cad=rja&uact=8&ved=0ahUKEwihsbGnoovPAhVBGJQKHfQwDV8QFggdMAA&url=https%3A%2F%2Fen.wikipedia.org%2Fwiki%2FBacillus_subtilis&usg=AFQjCNGFijWm9LtsdZ-Da63OlFj9u3JOdg&sig2=w2ehAcPeNMdxfmDxDgXppA&bvm=bv.132653024,d.dGo)  ATCC 14579 |
| 18 | × | × | ✓ | × | ✓ | ✓ | ✓ | [*E. coli*](https://www.google.co.kr/url?sa=t&rct=j&q=&esrc=s&source=web&cd=2&cad=rja&uact=8&ved=0ahUKEwi9reTfoYvPAhWGVZQKHa4iAs4QFggiMAE&url=https%3A%2F%2Fen.wikipedia.org%2Fwiki%2FEscherichia_coli&usg=AFQjCNFT_fJrAAEMjuPEQYfRdOqM5ifGCw&sig2=a3UiOKgWSQVOdmhHygmBEg)  0157: H7 ATCC 43890 |
| 19 | × | × | ✓ | × | ✓ | ✓ | ✓ | [*E. coli*](https://www.google.co.kr/url?sa=t&rct=j&q=&esrc=s&source=web&cd=2&cad=rja&uact=8&ved=0ahUKEwi9reTfoYvPAhWGVZQKHa4iAs4QFggiMAE&url=https%3A%2F%2Fen.wikipedia.org%2Fwiki%2FEscherichia_coli&usg=AFQjCNFT_fJrAAEMjuPEQYfRdOqM5ifGCw&sig2=a3UiOKgWSQVOdmhHygmBEg)  0157: H7 ATCC 43890 |
| 20 | ✓ | × | × | × | × | × | × | [*S.* *aureus*](https://www.google.co.kr/search?biw=1920&bih=934&q=staphylococcus+aureus&spell=1&sa=X&ved=0ahUKEwjTp_GboovPAhXEm5QKHYQJBgkQvwUIFygA) KCCM 40050 |
| 21 | × | ✓ | × | ✓ | × | ✓ | ✓ | [*B. subtilis*](https://www.google.co.kr/url?sa=t&rct=j&q=&esrc=s&source=web&cd=1&cad=rja&uact=8&ved=0ahUKEwihsbGnoovPAhVBGJQKHfQwDV8QFggdMAA&url=https%3A%2F%2Fen.wikipedia.org%2Fwiki%2FBacillus_subtilis&usg=AFQjCNGFijWm9LtsdZ-Da63OlFj9u3JOdg&sig2=w2ehAcPeNMdxfmDxDgXppA&bvm=bv.132653024,d.dGo)  ATCC 14579 |
| 22 | × | × | × | × | × | ✓ | ✓ | [*C. sakazakii*](https://www.google.co.kr/url?sa=t&rct=j&q=&esrc=s&source=web&cd=1&cad=rja&uact=8&ved=0ahUKEwjYuJDsoYvPAhXONpQKHUTkDh4QFggfMAA&url=https%3A%2F%2Fen.wikipedia.org%2Fwiki%2FCronobacter_sakazakii&usg=AFQjCNG4PxVztbOnpuuiy-BQnzqAysZdrw&sig2=jWhylTWYc9rD6Be3xz2CLQ)  ATCC 29004 |
| 23 | × | × | × | × | × | ✓ | ✓ | [*C. sakazakii*](https://www.google.co.kr/url?sa=t&rct=j&q=&esrc=s&source=web&cd=1&cad=rja&uact=8&ved=0ahUKEwjYuJDsoYvPAhXONpQKHUTkDh4QFggfMAA&url=https%3A%2F%2Fen.wikipedia.org%2Fwiki%2FCronobacter_sakazakii&usg=AFQjCNG4PxVztbOnpuuiy-BQnzqAysZdrw&sig2=jWhylTWYc9rD6Be3xz2CLQ)  ATCC 29004 |
| 24 | × | ✓ | × | ✓ | × | ✓ | ✓ | [*B. subtilis*](https://www.google.co.kr/url?sa=t&rct=j&q=&esrc=s&source=web&cd=1&cad=rja&uact=8&ved=0ahUKEwihsbGnoovPAhVBGJQKHfQwDV8QFggdMAA&url=https%3A%2F%2Fen.wikipedia.org%2Fwiki%2FBacillus_subtilis&usg=AFQjCNGFijWm9LtsdZ-Da63OlFj9u3JOdg&sig2=w2ehAcPeNMdxfmDxDgXppA&bvm=bv.132653024,d.dGo)  ATCC 14579 |
| 25 | × | × | ✓ | × | ✓ | ✓ | ✓ | Dead Cell |
| 26 | ✓ | × | × | × | ✓ | × | ✓ | [*L. monocytogenes*](https://www.google.co.kr/url?sa=t&rct=j&q=&esrc=s&source=web&cd=1&cad=rja&uact=8&ved=0ahUKEwjr1om9oovPAhWCoJQKHQXfAHcQFggiMAA&url=https%3A%2F%2Fen.wikipedia.org%2Fwiki%2FListeria_monocytogenes&usg=AFQjCNGclwOGCJ_vA7pTFhCwISnMtBS3kg&sig2=U-NJNe3xcw6TWk9ePvp35g) ATCC 19115 |
| 27 | × | × | ✓ | × | ✓ | ✓ | ✓ | [*S.* Typhimurium](https://www.google.co.kr/url?sa=t&rct=j&q=&esrc=s&source=web&cd=1&cad=rja&uact=8&ved=0ahUKEwiKhaH7oYvPAhVLnZQKHTqqB68QFgggMAA&url=https%3A%2F%2Fmicrobewiki.kenyon.edu%2Findex.php%2FSalmonella_typhimurium&usg=AFQjCNEPobR9rg-uDeUVgfriNNNDMuqFBA&sig2=KzrfANWPFUJSb-aM0n9-hw&bvm=bv.132653024,d.dGo)  ATCC 43971 |
| 28 | × | × | ✓ | × | ✓ | ✓ | ✓ | [*S.* Typhimurium](https://www.google.co.kr/url?sa=t&rct=j&q=&esrc=s&source=web&cd=1&cad=rja&uact=8&ved=0ahUKEwiKhaH7oYvPAhVLnZQKHTqqB68QFgggMAA&url=https%3A%2F%2Fmicrobewiki.kenyon.edu%2Findex.php%2FSalmonella_typhimurium&usg=AFQjCNEPobR9rg-uDeUVgfriNNNDMuqFBA&sig2=KzrfANWPFUJSb-aM0n9-hw&bvm=bv.132653024,d.dGo)  ATCC 43971 |
| 29 | ✓ | ✓ | × | ✓ | ✓ | ✓ | ✓ | [*L. monocytogenes*](https://www.google.co.kr/url?sa=t&rct=j&q=&esrc=s&source=web&cd=1&cad=rja&uact=8&ved=0ahUKEwjr1om9oovPAhWCoJQKHQXfAHcQFggiMAA&url=https%3A%2F%2Fen.wikipedia.org%2Fwiki%2FListeria_monocytogenes&usg=AFQjCNGclwOGCJ_vA7pTFhCwISnMtBS3kg&sig2=U-NJNe3xcw6TWk9ePvp35g) ATCC 19115 |
| 30 | ✓ | ✓ | × | ✓ | ✓ | ✓ | ✓ | [*L. monocytogenes*](https://www.google.co.kr/url?sa=t&rct=j&q=&esrc=s&source=web&cd=1&cad=rja&uact=8&ved=0ahUKEwjr1om9oovPAhWCoJQKHQXfAHcQFggiMAA&url=https%3A%2F%2Fen.wikipedia.org%2Fwiki%2FListeria_monocytogenes&usg=AFQjCNGclwOGCJ_vA7pTFhCwISnMtBS3kg&sig2=U-NJNe3xcw6TWk9ePvp35g) ATCC 19115 |
| 31 | × | × | × | × | × | ✓ | ✓ | [*C. sakazakii*](https://www.google.co.kr/url?sa=t&rct=j&q=&esrc=s&source=web&cd=1&cad=rja&uact=8&ved=0ahUKEwjYuJDsoYvPAhXONpQKHUTkDh4QFggfMAA&url=https%3A%2F%2Fen.wikipedia.org%2Fwiki%2FCronobacter_sakazakii&usg=AFQjCNG4PxVztbOnpuuiy-BQnzqAysZdrw&sig2=jWhylTWYc9rD6Be3xz2CLQ)  ATCC 29004 |
| 32 | × | × | ✓ | × | ✓ | ✓ | ✓ | [*S.* Typhimurium](https://www.google.co.kr/url?sa=t&rct=j&q=&esrc=s&source=web&cd=1&cad=rja&uact=8&ved=0ahUKEwiKhaH7oYvPAhVLnZQKHTqqB68QFgggMAA&url=https%3A%2F%2Fmicrobewiki.kenyon.edu%2Findex.php%2FSalmonella_typhimurium&usg=AFQjCNEPobR9rg-uDeUVgfriNNNDMuqFBA&sig2=KzrfANWPFUJSb-aM0n9-hw&bvm=bv.132653024,d.dGo)  ATCC 43971 |
| 33 | ✓ | ✓ | × | ✓ | ✓ | ✓ | ✓ | [*L. monocytogenes*](https://www.google.co.kr/url?sa=t&rct=j&q=&esrc=s&source=web&cd=1&cad=rja&uact=8&ved=0ahUKEwjr1om9oovPAhWCoJQKHQXfAHcQFggiMAA&url=https%3A%2F%2Fen.wikipedia.org%2Fwiki%2FListeria_monocytogenes&usg=AFQjCNGclwOGCJ_vA7pTFhCwISnMtBS3kg&sig2=U-NJNe3xcw6TWk9ePvp35g) ATCC 19115 |
| 34 | ✓ | ✓ | × | ✓ | ✓ | ✓ | ✓ | [*L. monocytogenes*](https://www.google.co.kr/url?sa=t&rct=j&q=&esrc=s&source=web&cd=1&cad=rja&uact=8&ved=0ahUKEwjr1om9oovPAhWCoJQKHQXfAHcQFggiMAA&url=https%3A%2F%2Fen.wikipedia.org%2Fwiki%2FListeria_monocytogenes&usg=AFQjCNGclwOGCJ_vA7pTFhCwISnMtBS3kg&sig2=U-NJNe3xcw6TWk9ePvp35g) ATCC 19115 |
| 35 | × | ✓ | × | ✓ | × | ✓ | ✓ | [*B. subtilis*](https://www.google.co.kr/url?sa=t&rct=j&q=&esrc=s&source=web&cd=1&cad=rja&uact=8&ved=0ahUKEwihsbGnoovPAhVBGJQKHfQwDV8QFggdMAA&url=https%3A%2F%2Fen.wikipedia.org%2Fwiki%2FBacillus_subtilis&usg=AFQjCNGFijWm9LtsdZ-Da63OlFj9u3JOdg&sig2=w2ehAcPeNMdxfmDxDgXppA&bvm=bv.132653024,d.dGo)  ATCC 14579 |
| 36 | ✓ | × | × | × | ✓ | × | ✓ | Debris |
| 37 | × | × | ✓ | × | ✓ | ✓ | ✓ | [*E. coli*](https://www.google.co.kr/url?sa=t&rct=j&q=&esrc=s&source=web&cd=2&cad=rja&uact=8&ved=0ahUKEwi9reTfoYvPAhWGVZQKHa4iAs4QFggiMAE&url=https%3A%2F%2Fen.wikipedia.org%2Fwiki%2FEscherichia_coli&usg=AFQjCNFT_fJrAAEMjuPEQYfRdOqM5ifGCw&sig2=a3UiOKgWSQVOdmhHygmBEg)  0157: H7 ATCC 43890 |
| 38 | ✓ | ✓ | ✓ | ✓ | ✓ | ✓ | ✓ | Dead Cell |
| 39 | × | × | ✓ | × | ✓ | ✓ | ✓ | Dead Cell |
| 40 | ✓ | ✓ | × | ✓ | ✓ | ✓ | ✓ | [*L. monocytogenes*](https://www.google.co.kr/url?sa=t&rct=j&q=&esrc=s&source=web&cd=1&cad=rja&uact=8&ved=0ahUKEwjr1om9oovPAhWCoJQKHQXfAHcQFggiMAA&url=https%3A%2F%2Fen.wikipedia.org%2Fwiki%2FListeria_monocytogenes&usg=AFQjCNGclwOGCJ_vA7pTFhCwISnMtBS3kg&sig2=U-NJNe3xcw6TWk9ePvp35g) ATCC 19115 |
| 41 | × | × | ✓ | × | ✓ | ✓ | ✓ | [*E. coli*](https://www.google.co.kr/url?sa=t&rct=j&q=&esrc=s&source=web&cd=2&cad=rja&uact=8&ved=0ahUKEwi9reTfoYvPAhWGVZQKHa4iAs4QFggiMAE&url=https%3A%2F%2Fen.wikipedia.org%2Fwiki%2FEscherichia_coli&usg=AFQjCNFT_fJrAAEMjuPEQYfRdOqM5ifGCw&sig2=a3UiOKgWSQVOdmhHygmBEg)  0157: H7 ATCC 43890 |
| 42 | × | ✓ | × | ✓ | × | ✓ | ✓ | [*B. subtilis*](https://www.google.co.kr/url?sa=t&rct=j&q=&esrc=s&source=web&cd=1&cad=rja&uact=8&ved=0ahUKEwihsbGnoovPAhVBGJQKHfQwDV8QFggdMAA&url=https%3A%2F%2Fen.wikipedia.org%2Fwiki%2FBacillus_subtilis&usg=AFQjCNGFijWm9LtsdZ-Da63OlFj9u3JOdg&sig2=w2ehAcPeNMdxfmDxDgXppA&bvm=bv.132653024,d.dGo)  ATCC 14579 |
| 43 | ✓ | ✓ | × | ✓ | ✓ | ✓ | ✓ | [*L. monocytogenes*](https://www.google.co.kr/url?sa=t&rct=j&q=&esrc=s&source=web&cd=1&cad=rja&uact=8&ved=0ahUKEwjr1om9oovPAhWCoJQKHQXfAHcQFggiMAA&url=https%3A%2F%2Fen.wikipedia.org%2Fwiki%2FListeria_monocytogenes&usg=AFQjCNGclwOGCJ_vA7pTFhCwISnMtBS3kg&sig2=U-NJNe3xcw6TWk9ePvp35g) ATCC 19115 |
| 44 | × | × | ✓ | × | ✓ | ✓ | ✓ | [*E. coli*](https://www.google.co.kr/url?sa=t&rct=j&q=&esrc=s&source=web&cd=2&cad=rja&uact=8&ved=0ahUKEwi9reTfoYvPAhWGVZQKHa4iAs4QFggiMAE&url=https%3A%2F%2Fen.wikipedia.org%2Fwiki%2FEscherichia_coli&usg=AFQjCNFT_fJrAAEMjuPEQYfRdOqM5ifGCw&sig2=a3UiOKgWSQVOdmhHygmBEg)  0157: H7 ATCC 43890 |
| 45 | × | × | × | × | × | ✓ | ✓ | [*C. sakazakii*](https://www.google.co.kr/url?sa=t&rct=j&q=&esrc=s&source=web&cd=1&cad=rja&uact=8&ved=0ahUKEwjYuJDsoYvPAhXONpQKHUTkDh4QFggfMAA&url=https%3A%2F%2Fen.wikipedia.org%2Fwiki%2FCronobacter_sakazakii&usg=AFQjCNG4PxVztbOnpuuiy-BQnzqAysZdrw&sig2=jWhylTWYc9rD6Be3xz2CLQ)  ATCC 29004 |
| 46 | × | × | ✓ | × | ✓ | ✓ | ✓ | Dead Cell |
| 47 | ✓ | ✓ | × | ✓ | ✓ | ✓ | ✓ | [*L. monocytogenes*](https://www.google.co.kr/url?sa=t&rct=j&q=&esrc=s&source=web&cd=1&cad=rja&uact=8&ved=0ahUKEwjr1om9oovPAhWCoJQKHQXfAHcQFggiMAA&url=https%3A%2F%2Fen.wikipedia.org%2Fwiki%2FListeria_monocytogenes&usg=AFQjCNGclwOGCJ_vA7pTFhCwISnMtBS3kg&sig2=U-NJNe3xcw6TWk9ePvp35g) ATCC 19115 |
| 48 | × | × | × | × | × | ✓ | ✓ | [*C. sakazakii*](https://www.google.co.kr/url?sa=t&rct=j&q=&esrc=s&source=web&cd=1&cad=rja&uact=8&ved=0ahUKEwjYuJDsoYvPAhXONpQKHUTkDh4QFggfMAA&url=https%3A%2F%2Fen.wikipedia.org%2Fwiki%2FCronobacter_sakazakii&usg=AFQjCNG4PxVztbOnpuuiy-BQnzqAysZdrw&sig2=jWhylTWYc9rD6Be3xz2CLQ)  ATCC 29004 |
| 49 | × | × | ✓ | × | ✓ | ✓ | ✓ | [*S.* Typhimurium](https://www.google.co.kr/url?sa=t&rct=j&q=&esrc=s&source=web&cd=1&cad=rja&uact=8&ved=0ahUKEwiKhaH7oYvPAhVLnZQKHTqqB68QFgggMAA&url=https%3A%2F%2Fmicrobewiki.kenyon.edu%2Findex.php%2FSalmonella_typhimurium&usg=AFQjCNEPobR9rg-uDeUVgfriNNNDMuqFBA&sig2=KzrfANWPFUJSb-aM0n9-hw&bvm=bv.132653024,d.dGo)  ATCC 43971 |
| 50 | × | × | ✓ | × | ✓ | ✓ | ✓ | [*S.* Typhimurium](https://www.google.co.kr/url?sa=t&rct=j&q=&esrc=s&source=web&cd=1&cad=rja&uact=8&ved=0ahUKEwiKhaH7oYvPAhVLnZQKHTqqB68QFgggMAA&url=https%3A%2F%2Fmicrobewiki.kenyon.edu%2Findex.php%2FSalmonella_typhimurium&usg=AFQjCNEPobR9rg-uDeUVgfriNNNDMuqFBA&sig2=KzrfANWPFUJSb-aM0n9-hw&bvm=bv.132653024,d.dGo)  ATCC 43971 |
| 51 | ✓ | ✓ | × | ✓ | ✓ | ✓ | ✓ | [*L. monocytogenes*](https://www.google.co.kr/url?sa=t&rct=j&q=&esrc=s&source=web&cd=1&cad=rja&uact=8&ved=0ahUKEwjr1om9oovPAhWCoJQKHQXfAHcQFggiMAA&url=https%3A%2F%2Fen.wikipedia.org%2Fwiki%2FListeria_monocytogenes&usg=AFQjCNGclwOGCJ_vA7pTFhCwISnMtBS3kg&sig2=U-NJNe3xcw6TWk9ePvp35g) ATCC 19115 |
| 52 | × | × | ✓ | × | ✓ | ✓ | ✓ | Dead Cell |
| 53 | × | ✓ | × | ✓ | × | ✓ | ✓ | [*B. subtilis*](https://www.google.co.kr/url?sa=t&rct=j&q=&esrc=s&source=web&cd=1&cad=rja&uact=8&ved=0ahUKEwihsbGnoovPAhVBGJQKHfQwDV8QFggdMAA&url=https%3A%2F%2Fen.wikipedia.org%2Fwiki%2FBacillus_subtilis&usg=AFQjCNGFijWm9LtsdZ-Da63OlFj9u3JOdg&sig2=w2ehAcPeNMdxfmDxDgXppA&bvm=bv.132653024,d.dGo)  ATCC 14579 |
| 54 | × | × | ✓ | × | ✓ | ✓ | ✓ | [*E. coli*](https://www.google.co.kr/url?sa=t&rct=j&q=&esrc=s&source=web&cd=2&cad=rja&uact=8&ved=0ahUKEwi9reTfoYvPAhWGVZQKHa4iAs4QFggiMAE&url=https%3A%2F%2Fen.wikipedia.org%2Fwiki%2FEscherichia_coli&usg=AFQjCNFT_fJrAAEMjuPEQYfRdOqM5ifGCw&sig2=a3UiOKgWSQVOdmhHygmBEg)  0157: H7 ATCC 43890 |
| 55 | ✓ | ✓ | × | ✓ | ✓ | ✓ | ✓ | [*L. monocytogenes*](https://www.google.co.kr/url?sa=t&rct=j&q=&esrc=s&source=web&cd=1&cad=rja&uact=8&ved=0ahUKEwjr1om9oovPAhWCoJQKHQXfAHcQFggiMAA&url=https%3A%2F%2Fen.wikipedia.org%2Fwiki%2FListeria_monocytogenes&usg=AFQjCNGclwOGCJ_vA7pTFhCwISnMtBS3kg&sig2=U-NJNe3xcw6TWk9ePvp35g) ATCC 19115 |
| 56 | × | × | × | × | × | ✓ | ✓ | [*C. sakazakii*](https://www.google.co.kr/url?sa=t&rct=j&q=&esrc=s&source=web&cd=1&cad=rja&uact=8&ved=0ahUKEwjYuJDsoYvPAhXONpQKHUTkDh4QFggfMAA&url=https%3A%2F%2Fen.wikipedia.org%2Fwiki%2FCronobacter_sakazakii&usg=AFQjCNG4PxVztbOnpuuiy-BQnzqAysZdrw&sig2=jWhylTWYc9rD6Be3xz2CLQ)  ATCC 29004 |
| 57 | × | × | ✓ | × | ✓ | ✓ | ✓ | [*E. coli*](https://www.google.co.kr/url?sa=t&rct=j&q=&esrc=s&source=web&cd=2&cad=rja&uact=8&ved=0ahUKEwi9reTfoYvPAhWGVZQKHa4iAs4QFggiMAE&url=https%3A%2F%2Fen.wikipedia.org%2Fwiki%2FEscherichia_coli&usg=AFQjCNFT_fJrAAEMjuPEQYfRdOqM5ifGCw&sig2=a3UiOKgWSQVOdmhHygmBEg)  0157: H7 ATCC 43890 |
| 58 | × | × | × | × | × | ✓ | ✓ | [*C. sakazakii*](https://www.google.co.kr/url?sa=t&rct=j&q=&esrc=s&source=web&cd=1&cad=rja&uact=8&ved=0ahUKEwjYuJDsoYvPAhXONpQKHUTkDh4QFggfMAA&url=https%3A%2F%2Fen.wikipedia.org%2Fwiki%2FCronobacter_sakazakii&usg=AFQjCNG4PxVztbOnpuuiy-BQnzqAysZdrw&sig2=jWhylTWYc9rD6Be3xz2CLQ)  ATCC 29004 |
| 59 | ✓ | ✓ | × | ✓ | ✓ | ✓ | ✓ | [*L. monocytogenes*](https://www.google.co.kr/url?sa=t&rct=j&q=&esrc=s&source=web&cd=1&cad=rja&uact=8&ved=0ahUKEwjr1om9oovPAhWCoJQKHQXfAHcQFggiMAA&url=https%3A%2F%2Fen.wikipedia.org%2Fwiki%2FListeria_monocytogenes&usg=AFQjCNGclwOGCJ_vA7pTFhCwISnMtBS3kg&sig2=U-NJNe3xcw6TWk9ePvp35g) ATCC 19115 |
| 60 | × | × | ✓ | × | ✓ | ✓ | ✓ | Dead Cell |
| 61 | × | × | × | × | × | × | ✓ | [*S.* *aureus*](https://www.google.co.kr/search?biw=1920&bih=934&q=staphylococcus+aureus&spell=1&sa=X&ved=0ahUKEwjTp_GboovPAhXEm5QKHYQJBgkQvwUIFygA) KCCM 40050 |
| 62 | × | × | ✓ | × | ✓ | ✓ | ✓ | [*E. coli*](https://www.google.co.kr/url?sa=t&rct=j&q=&esrc=s&source=web&cd=2&cad=rja&uact=8&ved=0ahUKEwi9reTfoYvPAhWGVZQKHa4iAs4QFggiMAE&url=https%3A%2F%2Fen.wikipedia.org%2Fwiki%2FEscherichia_coli&usg=AFQjCNFT_fJrAAEMjuPEQYfRdOqM5ifGCw&sig2=a3UiOKgWSQVOdmhHygmBEg)  0157: H7 ATCC 43890 |
| 63 | × | × | ✓ | × | ✓ | ✓ | ✓ | Dead Cell |
| 64 | ✓ | ✓ | × | ✓ | ✓ | ✓ | ✓ | [*L. monocytogenes*](https://www.google.co.kr/url?sa=t&rct=j&q=&esrc=s&source=web&cd=1&cad=rja&uact=8&ved=0ahUKEwjr1om9oovPAhWCoJQKHQXfAHcQFggiMAA&url=https%3A%2F%2Fen.wikipedia.org%2Fwiki%2FListeria_monocytogenes&usg=AFQjCNGclwOGCJ_vA7pTFhCwISnMtBS3kg&sig2=U-NJNe3xcw6TWk9ePvp35g) ATCC 19115 |
| 65 | × | × | × | × | × | ✓ | ✓ | [*C. sakazakii*](https://www.google.co.kr/url?sa=t&rct=j&q=&esrc=s&source=web&cd=1&cad=rja&uact=8&ved=0ahUKEwjYuJDsoYvPAhXONpQKHUTkDh4QFggfMAA&url=https%3A%2F%2Fen.wikipedia.org%2Fwiki%2FCronobacter_sakazakii&usg=AFQjCNG4PxVztbOnpuuiy-BQnzqAysZdrw&sig2=jWhylTWYc9rD6Be3xz2CLQ)  ATCC 29004 |
| 66 | × | × | ✓ | × | ✓ | ✓ | ✓ | Dead Cell |
| 67 | × | ✓ | × | ✓ | × | ✓ | ✓ | [*B. subtilis*](https://www.google.co.kr/url?sa=t&rct=j&q=&esrc=s&source=web&cd=1&cad=rja&uact=8&ved=0ahUKEwihsbGnoovPAhVBGJQKHfQwDV8QFggdMAA&url=https%3A%2F%2Fen.wikipedia.org%2Fwiki%2FBacillus_subtilis&usg=AFQjCNGFijWm9LtsdZ-Da63OlFj9u3JOdg&sig2=w2ehAcPeNMdxfmDxDgXppA&bvm=bv.132653024,d.dGo)  ATCC 14579 |
| 68 | ✓ | ✓ | × | ✓ | ✓ | ✓ | ✓ | [*L. monocytogenes*](https://www.google.co.kr/url?sa=t&rct=j&q=&esrc=s&source=web&cd=1&cad=rja&uact=8&ved=0ahUKEwjr1om9oovPAhWCoJQKHQXfAHcQFggiMAA&url=https%3A%2F%2Fen.wikipedia.org%2Fwiki%2FListeria_monocytogenes&usg=AFQjCNGclwOGCJ_vA7pTFhCwISnMtBS3kg&sig2=U-NJNe3xcw6TWk9ePvp35g) ATCC 19115 |
| 69 | ✓ | × | × | × | ✓ | × | ✓ | [*L. monocytogenes*](https://www.google.co.kr/url?sa=t&rct=j&q=&esrc=s&source=web&cd=1&cad=rja&uact=8&ved=0ahUKEwjr1om9oovPAhWCoJQKHQXfAHcQFggiMAA&url=https%3A%2F%2Fen.wikipedia.org%2Fwiki%2FListeria_monocytogenes&usg=AFQjCNGclwOGCJ_vA7pTFhCwISnMtBS3kg&sig2=U-NJNe3xcw6TWk9ePvp35g) ATCC 19115 |
| 70 | ✓ | × | × | × | ✓ | × | ✓ | [*L. monocytogenes*](https://www.google.co.kr/url?sa=t&rct=j&q=&esrc=s&source=web&cd=1&cad=rja&uact=8&ved=0ahUKEwjr1om9oovPAhWCoJQKHQXfAHcQFggiMAA&url=https%3A%2F%2Fen.wikipedia.org%2Fwiki%2FListeria_monocytogenes&usg=AFQjCNGclwOGCJ_vA7pTFhCwISnMtBS3kg&sig2=U-NJNe3xcw6TWk9ePvp35g) ATCC 19115 |
| 71 | × | ✓ | × | ✓ | × | ✓ | ✓ | [*B. subtilis*](https://www.google.co.kr/url?sa=t&rct=j&q=&esrc=s&source=web&cd=1&cad=rja&uact=8&ved=0ahUKEwihsbGnoovPAhVBGJQKHfQwDV8QFggdMAA&url=https%3A%2F%2Fen.wikipedia.org%2Fwiki%2FBacillus_subtilis&usg=AFQjCNGFijWm9LtsdZ-Da63OlFj9u3JOdg&sig2=w2ehAcPeNMdxfmDxDgXppA&bvm=bv.132653024,d.dGo)  ATCC 14579 |
| 72 | × | × | × | × | × | ✓ | ✓ | [*C. sakazakii*](https://www.google.co.kr/url?sa=t&rct=j&q=&esrc=s&source=web&cd=1&cad=rja&uact=8&ved=0ahUKEwjYuJDsoYvPAhXONpQKHUTkDh4QFggfMAA&url=https%3A%2F%2Fen.wikipedia.org%2Fwiki%2FCronobacter_sakazakii&usg=AFQjCNG4PxVztbOnpuuiy-BQnzqAysZdrw&sig2=jWhylTWYc9rD6Be3xz2CLQ)  ATCC 29004 |
| 73 | × | × | × | × | × | ✓ | ✓ | [*C. sakazakii*](https://www.google.co.kr/url?sa=t&rct=j&q=&esrc=s&source=web&cd=1&cad=rja&uact=8&ved=0ahUKEwjYuJDsoYvPAhXONpQKHUTkDh4QFggfMAA&url=https%3A%2F%2Fen.wikipedia.org%2Fwiki%2FCronobacter_sakazakii&usg=AFQjCNG4PxVztbOnpuuiy-BQnzqAysZdrw&sig2=jWhylTWYc9rD6Be3xz2CLQ)  ATCC 29004 |
| 74 | × | × | × | × | × | × | ✓ | [*L. monocytogenes*](https://www.google.co.kr/url?sa=t&rct=j&q=&esrc=s&source=web&cd=1&cad=rja&uact=8&ved=0ahUKEwjr1om9oovPAhWCoJQKHQXfAHcQFggiMAA&url=https%3A%2F%2Fen.wikipedia.org%2Fwiki%2FListeria_monocytogenes&usg=AFQjCNGclwOGCJ_vA7pTFhCwISnMtBS3kg&sig2=U-NJNe3xcw6TWk9ePvp35g) ATCC 19115 |
| 75 | × | × | ✓ | × | ✓ | ✓ | ✓ | [*S.* Typhimurium](https://www.google.co.kr/url?sa=t&rct=j&q=&esrc=s&source=web&cd=1&cad=rja&uact=8&ved=0ahUKEwiKhaH7oYvPAhVLnZQKHTqqB68QFgggMAA&url=https%3A%2F%2Fmicrobewiki.kenyon.edu%2Findex.php%2FSalmonella_typhimurium&usg=AFQjCNEPobR9rg-uDeUVgfriNNNDMuqFBA&sig2=KzrfANWPFUJSb-aM0n9-hw&bvm=bv.132653024,d.dGo)  ATCC 43971 |
| 76 | ✓ | × | ✓ | ✓ | ✓ | ✓ | ✓ | Injured cell |
| 77 | × | × | ✓ | × | ✓ | ✓ | ✓ | Dead Cell |
| 78 | ✓ | ✓ | × | ✓ | ✓ | ✓ | ✓ | [*L. monocytogenes*](https://www.google.co.kr/url?sa=t&rct=j&q=&esrc=s&source=web&cd=1&cad=rja&uact=8&ved=0ahUKEwjr1om9oovPAhWCoJQKHQXfAHcQFggiMAA&url=https%3A%2F%2Fen.wikipedia.org%2Fwiki%2FListeria_monocytogenes&usg=AFQjCNGclwOGCJ_vA7pTFhCwISnMtBS3kg&sig2=U-NJNe3xcw6TWk9ePvp35g) ATCC 19115 |
| 79 | × | × | ✓ | × | ✓ | ✓ | ✓ | Dead Cell |
| 80 | × | ✓ | × | ✓ | × | ✓ | ✓ | [*B. subtilis*](https://www.google.co.kr/url?sa=t&rct=j&q=&esrc=s&source=web&cd=1&cad=rja&uact=8&ved=0ahUKEwihsbGnoovPAhVBGJQKHfQwDV8QFggdMAA&url=https%3A%2F%2Fen.wikipedia.org%2Fwiki%2FBacillus_subtilis&usg=AFQjCNGFijWm9LtsdZ-Da63OlFj9u3JOdg&sig2=w2ehAcPeNMdxfmDxDgXppA&bvm=bv.132653024,d.dGo)  ATCC 14579 |
| 81 | ✓ | × | × | × | × | × | × | [*S.* *aureus*](https://www.google.co.kr/search?biw=1920&bih=934&q=staphylococcus+aureus&spell=1&sa=X&ved=0ahUKEwjTp_GboovPAhXEm5QKHYQJBgkQvwUIFygA) KCCM 40050 |
| 82 | × | ✓ | × | ✓ | × | ✓ | ✓ | [*B. subtilis*](https://www.google.co.kr/url?sa=t&rct=j&q=&esrc=s&source=web&cd=1&cad=rja&uact=8&ved=0ahUKEwihsbGnoovPAhVBGJQKHfQwDV8QFggdMAA&url=https%3A%2F%2Fen.wikipedia.org%2Fwiki%2FBacillus_subtilis&usg=AFQjCNGFijWm9LtsdZ-Da63OlFj9u3JOdg&sig2=w2ehAcPeNMdxfmDxDgXppA&bvm=bv.132653024,d.dGo)  ATCC 14579 |
| 83 | × | × | ✓ | × | ✓ | ✓ | ✓ | [*E. coli*](https://www.google.co.kr/url?sa=t&rct=j&q=&esrc=s&source=web&cd=2&cad=rja&uact=8&ved=0ahUKEwi9reTfoYvPAhWGVZQKHa4iAs4QFggiMAE&url=https%3A%2F%2Fen.wikipedia.org%2Fwiki%2FEscherichia_coli&usg=AFQjCNFT_fJrAAEMjuPEQYfRdOqM5ifGCw&sig2=a3UiOKgWSQVOdmhHygmBEg)  0157: H7 ATCC 43890 |
| 84 | ✓ | × | × | × | × | × | × | [*S.* *aureus*](https://www.google.co.kr/search?biw=1920&bih=934&q=staphylococcus+aureus&spell=1&sa=X&ved=0ahUKEwjTp_GboovPAhXEm5QKHYQJBgkQvwUIFygA) KCCM 40050 |
| 85 | × | × | ✓ | × | ✓ | ✓ | ✓ | Dead Cell |
| 86 | ✓ | × | ✓ | ✓ | ✓ | ✓ | ✓ | Injured cell |
| 87 | × | × | ✓ | × | ✓ | ✓ | ✓ | Dead Cell |
| 88 | × | × | ✓ | × | ✓ | ✓ | ✓ | Dead Cell |
| 89 | ✓ | × | ✓ | ✓ | ✓ | ✓ | ✓ | Injured cell |
| 90 | × | ✓ | × | ✓ | × | ✓ | ✓ | [*B. subtilis*](https://www.google.co.kr/url?sa=t&rct=j&q=&esrc=s&source=web&cd=1&cad=rja&uact=8&ved=0ahUKEwihsbGnoovPAhVBGJQKHfQwDV8QFggdMAA&url=https%3A%2F%2Fen.wikipedia.org%2Fwiki%2FBacillus_subtilis&usg=AFQjCNGFijWm9LtsdZ-Da63OlFj9u3JOdg&sig2=w2ehAcPeNMdxfmDxDgXppA&bvm=bv.132653024,d.dGo)  ATCC 14579 |
| 91 | ✓ | ✓ | ✓ | ✓ | ✓ | ✓ | ✓ | Dead Cell |
| 92 | × | × | ✓ | × | ✓ | ✓ | ✓ | [*S.* Typhimurium](https://www.google.co.kr/url?sa=t&rct=j&q=&esrc=s&source=web&cd=1&cad=rja&uact=8&ved=0ahUKEwiKhaH7oYvPAhVLnZQKHTqqB68QFgggMAA&url=https%3A%2F%2Fmicrobewiki.kenyon.edu%2Findex.php%2FSalmonella_typhimurium&usg=AFQjCNEPobR9rg-uDeUVgfriNNNDMuqFBA&sig2=KzrfANWPFUJSb-aM0n9-hw&bvm=bv.132653024,d.dGo)  ATCC 43971 |
| 93 | ✓ | ✓ | × | ✓ | ✓ | ✓ | ✓ | [*L. monocytogenes*](https://www.google.co.kr/url?sa=t&rct=j&q=&esrc=s&source=web&cd=1&cad=rja&uact=8&ved=0ahUKEwjr1om9oovPAhWCoJQKHQXfAHcQFggiMAA&url=https%3A%2F%2Fen.wikipedia.org%2Fwiki%2FListeria_monocytogenes&usg=AFQjCNGclwOGCJ_vA7pTFhCwISnMtBS3kg&sig2=U-NJNe3xcw6TWk9ePvp35g) ATCC 19115 |
| 94 | × | × | ✓ | × | ✓ | ✓ | ✓ | [*S.* Typhimurium](https://www.google.co.kr/url?sa=t&rct=j&q=&esrc=s&source=web&cd=1&cad=rja&uact=8&ved=0ahUKEwiKhaH7oYvPAhVLnZQKHTqqB68QFgggMAA&url=https%3A%2F%2Fmicrobewiki.kenyon.edu%2Findex.php%2FSalmonella_typhimurium&usg=AFQjCNEPobR9rg-uDeUVgfriNNNDMuqFBA&sig2=KzrfANWPFUJSb-aM0n9-hw&bvm=bv.132653024,d.dGo)  ATCC 43971 |
| 95 | × | × | × | × | × | × | ✓ | [*L. monocytogenes*](https://www.google.co.kr/url?sa=t&rct=j&q=&esrc=s&source=web&cd=1&cad=rja&uact=8&ved=0ahUKEwjr1om9oovPAhWCoJQKHQXfAHcQFggiMAA&url=https%3A%2F%2Fen.wikipedia.org%2Fwiki%2FListeria_monocytogenes&usg=AFQjCNGclwOGCJ_vA7pTFhCwISnMtBS3kg&sig2=U-NJNe3xcw6TWk9ePvp35g) ATCC 19115 |
| 96 | ✓ | × | × | × | × | × | × | [*S.* *aureus*](https://www.google.co.kr/search?biw=1920&bih=934&q=staphylococcus+aureus&spell=1&sa=X&ved=0ahUKEwjTp_GboovPAhXEm5QKHYQJBgkQvwUIFygA) KCCM 40050 |
| 97 | × | × | × | × | × | × | ✓ | [*L. monocytogenes*](https://www.google.co.kr/url?sa=t&rct=j&q=&esrc=s&source=web&cd=1&cad=rja&uact=8&ved=0ahUKEwjr1om9oovPAhWCoJQKHQXfAHcQFggiMAA&url=https%3A%2F%2Fen.wikipedia.org%2Fwiki%2FListeria_monocytogenes&usg=AFQjCNGclwOGCJ_vA7pTFhCwISnMtBS3kg&sig2=U-NJNe3xcw6TWk9ePvp35g) ATCC 19115 |
| 98 | ✓ | ✓ | ✓ | ✓ | ✓ | ✓ | ✓ | Dead cell |
| 99 | × | × | ✓ | × | ✓ | ✓ | ✓ | [*E. coli*](https://www.google.co.kr/url?sa=t&rct=j&q=&esrc=s&source=web&cd=2&cad=rja&uact=8&ved=0ahUKEwi9reTfoYvPAhWGVZQKHa4iAs4QFggiMAE&url=https%3A%2F%2Fen.wikipedia.org%2Fwiki%2FEscherichia_coli&usg=AFQjCNFT_fJrAAEMjuPEQYfRdOqM5ifGCw&sig2=a3UiOKgWSQVOdmhHygmBEg)  0157: H7 ATCC 43890 |
| 100 | ✓ | ✓ | × | ✓ | ✓ | ✓ | ✓ | [*L. monocytogenes*](https://www.google.co.kr/url?sa=t&rct=j&q=&esrc=s&source=web&cd=1&cad=rja&uact=8&ved=0ahUKEwjr1om9oovPAhWCoJQKHQXfAHcQFggiMAA&url=https%3A%2F%2Fen.wikipedia.org%2Fwiki%2FListeria_monocytogenes&usg=AFQjCNGclwOGCJ_vA7pTFhCwISnMtBS3kg&sig2=U-NJNe3xcw6TWk9ePvp35g) ATCC 19115 |
| 101 | ✓ | ✓ | × | ✓ | ✓ | ✓ | ✓ | [*L. monocytogenes*](https://www.google.co.kr/url?sa=t&rct=j&q=&esrc=s&source=web&cd=1&cad=rja&uact=8&ved=0ahUKEwjr1om9oovPAhWCoJQKHQXfAHcQFggiMAA&url=https%3A%2F%2Fen.wikipedia.org%2Fwiki%2FListeria_monocytogenes&usg=AFQjCNGclwOGCJ_vA7pTFhCwISnMtBS3kg&sig2=U-NJNe3xcw6TWk9ePvp35g) ATCC 19115 |
| 102 | × | × | ✓ | × | ✓ | ✓ | ✓ | [*E. coli*](https://www.google.co.kr/url?sa=t&rct=j&q=&esrc=s&source=web&cd=2&cad=rja&uact=8&ved=0ahUKEwi9reTfoYvPAhWGVZQKHa4iAs4QFggiMAE&url=https%3A%2F%2Fen.wikipedia.org%2Fwiki%2FEscherichia_coli&usg=AFQjCNFT_fJrAAEMjuPEQYfRdOqM5ifGCw&sig2=a3UiOKgWSQVOdmhHygmBEg)  0157: H7 ATCC 43890 |
| 103 | ✓ | ✓ | × | ✓ | ✓ | ✓ | ✓ | [*L. monocytogenes*](https://www.google.co.kr/url?sa=t&rct=j&q=&esrc=s&source=web&cd=1&cad=rja&uact=8&ved=0ahUKEwjr1om9oovPAhWCoJQKHQXfAHcQFggiMAA&url=https%3A%2F%2Fen.wikipedia.org%2Fwiki%2FListeria_monocytogenes&usg=AFQjCNGclwOGCJ_vA7pTFhCwISnMtBS3kg&sig2=U-NJNe3xcw6TWk9ePvp35g) ATCC 19115 |
| 104 | ✓ | × | × | × | × | × | × | [*S.* *aureus*](https://www.google.co.kr/search?biw=1920&bih=934&q=staphylococcus+aureus&spell=1&sa=X&ved=0ahUKEwjTp_GboovPAhXEm5QKHYQJBgkQvwUIFygA) KCCM 40050 |
| 105 | ✓ | ✓ | × | ✓ | ✓ | ✓ | ✓ | [*L. monocytogenes*](https://www.google.co.kr/url?sa=t&rct=j&q=&esrc=s&source=web&cd=1&cad=rja&uact=8&ved=0ahUKEwjr1om9oovPAhWCoJQKHQXfAHcQFggiMAA&url=https%3A%2F%2Fen.wikipedia.org%2Fwiki%2FListeria_monocytogenes&usg=AFQjCNGclwOGCJ_vA7pTFhCwISnMtBS3kg&sig2=U-NJNe3xcw6TWk9ePvp35g) ATCC 19115 |
| 106 | ✓ | × | × | × | × | × | × | [*S.* *aureus*](https://www.google.co.kr/search?biw=1920&bih=934&q=staphylococcus+aureus&spell=1&sa=X&ved=0ahUKEwjTp_GboovPAhXEm5QKHYQJBgkQvwUIFygA) KCCM 40050 |
| 107 | × | ✓ | × | ✓ | × | ✓ | ✓ | [*B. subtilis*](https://www.google.co.kr/url?sa=t&rct=j&q=&esrc=s&source=web&cd=1&cad=rja&uact=8&ved=0ahUKEwihsbGnoovPAhVBGJQKHfQwDV8QFggdMAA&url=https%3A%2F%2Fen.wikipedia.org%2Fwiki%2FBacillus_subtilis&usg=AFQjCNGFijWm9LtsdZ-Da63OlFj9u3JOdg&sig2=w2ehAcPeNMdxfmDxDgXppA&bvm=bv.132653024,d.dGo)  ATCC 14579 |
| 108 | × | × | × | × | × | ✓ | ✓ | [*C. sakazakii*](https://www.google.co.kr/url?sa=t&rct=j&q=&esrc=s&source=web&cd=1&cad=rja&uact=8&ved=0ahUKEwjYuJDsoYvPAhXONpQKHUTkDh4QFggfMAA&url=https%3A%2F%2Fen.wikipedia.org%2Fwiki%2FCronobacter_sakazakii&usg=AFQjCNG4PxVztbOnpuuiy-BQnzqAysZdrw&sig2=jWhylTWYc9rD6Be3xz2CLQ)  ATCC 29004 |
| 109 | × | × | ✓ | × | ✓ | ✓ | ✓ | [*E. coli*](https://www.google.co.kr/url?sa=t&rct=j&q=&esrc=s&source=web&cd=2&cad=rja&uact=8&ved=0ahUKEwi9reTfoYvPAhWGVZQKHa4iAs4QFggiMAE&url=https%3A%2F%2Fen.wikipedia.org%2Fwiki%2FEscherichia_coli&usg=AFQjCNFT_fJrAAEMjuPEQYfRdOqM5ifGCw&sig2=a3UiOKgWSQVOdmhHygmBEg)  0157: H7 ATCC 43890 |
| 110 | × | × | × | × | × | × | ✓ | [*L. monocytogenes*](https://www.google.co.kr/url?sa=t&rct=j&q=&esrc=s&source=web&cd=1&cad=rja&uact=8&ved=0ahUKEwjr1om9oovPAhWCoJQKHQXfAHcQFggiMAA&url=https%3A%2F%2Fen.wikipedia.org%2Fwiki%2FListeria_monocytogenes&usg=AFQjCNGclwOGCJ_vA7pTFhCwISnMtBS3kg&sig2=U-NJNe3xcw6TWk9ePvp35g) ATCC 19115 |
| 111 | × | ✓ | × | ✓ | × | ✓ | ✓ | [*B. subtilis*](https://www.google.co.kr/url?sa=t&rct=j&q=&esrc=s&source=web&cd=1&cad=rja&uact=8&ved=0ahUKEwihsbGnoovPAhVBGJQKHfQwDV8QFggdMAA&url=https%3A%2F%2Fen.wikipedia.org%2Fwiki%2FBacillus_subtilis&usg=AFQjCNGFijWm9LtsdZ-Da63OlFj9u3JOdg&sig2=w2ehAcPeNMdxfmDxDgXppA&bvm=bv.132653024,d.dGo)  ATCC 14579 |
| 112 | × | × | ✓ | × | ✓ | ✓ | ✓ | Dead Cell |
| 113 | × | ✓ | × | ✓ | × | ✓ | ✓ | [*B. subtilis*](https://www.google.co.kr/url?sa=t&rct=j&q=&esrc=s&source=web&cd=1&cad=rja&uact=8&ved=0ahUKEwihsbGnoovPAhVBGJQKHfQwDV8QFggdMAA&url=https%3A%2F%2Fen.wikipedia.org%2Fwiki%2FBacillus_subtilis&usg=AFQjCNGFijWm9LtsdZ-Da63OlFj9u3JOdg&sig2=w2ehAcPeNMdxfmDxDgXppA&bvm=bv.132653024,d.dGo)  ATCC 14579 |
| 114 | × | ✓ | × | ✓ | × | ✓ | ✓ | [*B. subtilis*](https://www.google.co.kr/url?sa=t&rct=j&q=&esrc=s&source=web&cd=1&cad=rja&uact=8&ved=0ahUKEwihsbGnoovPAhVBGJQKHfQwDV8QFggdMAA&url=https%3A%2F%2Fen.wikipedia.org%2Fwiki%2FBacillus_subtilis&usg=AFQjCNGFijWm9LtsdZ-Da63OlFj9u3JOdg&sig2=w2ehAcPeNMdxfmDxDgXppA&bvm=bv.132653024,d.dGo)  ATCC 14579 |
| 115 | ✓ | ✓ | × | ✓ | ✓ | ✓ | ✓ | [*L. monocytogenes*](https://www.google.co.kr/url?sa=t&rct=j&q=&esrc=s&source=web&cd=1&cad=rja&uact=8&ved=0ahUKEwjr1om9oovPAhWCoJQKHQXfAHcQFggiMAA&url=https%3A%2F%2Fen.wikipedia.org%2Fwiki%2FListeria_monocytogenes&usg=AFQjCNGclwOGCJ_vA7pTFhCwISnMtBS3kg&sig2=U-NJNe3xcw6TWk9ePvp35g) ATCC 19115 |
| 116 | × | ✓ | × | ✓ | × | ✓ | ✓ | [*B. subtilis*](https://www.google.co.kr/url?sa=t&rct=j&q=&esrc=s&source=web&cd=1&cad=rja&uact=8&ved=0ahUKEwihsbGnoovPAhVBGJQKHfQwDV8QFggdMAA&url=https%3A%2F%2Fen.wikipedia.org%2Fwiki%2FBacillus_subtilis&usg=AFQjCNGFijWm9LtsdZ-Da63OlFj9u3JOdg&sig2=w2ehAcPeNMdxfmDxDgXppA&bvm=bv.132653024,d.dGo)  ATCC 14579 |
| 117 | ✓ | ✓ | × | ✓ | ✓ | ✓ | ✓ | [*L. monocytogenes*](https://www.google.co.kr/url?sa=t&rct=j&q=&esrc=s&source=web&cd=1&cad=rja&uact=8&ved=0ahUKEwjr1om9oovPAhWCoJQKHQXfAHcQFggiMAA&url=https%3A%2F%2Fen.wikipedia.org%2Fwiki%2FListeria_monocytogenes&usg=AFQjCNGclwOGCJ_vA7pTFhCwISnMtBS3kg&sig2=U-NJNe3xcw6TWk9ePvp35g) ATCC 19115 |
| 118 | × | × | ✓ | × | ✓ | ✓ | ✓ | [*E. coli*](https://www.google.co.kr/url?sa=t&rct=j&q=&esrc=s&source=web&cd=2&cad=rja&uact=8&ved=0ahUKEwi9reTfoYvPAhWGVZQKHa4iAs4QFggiMAE&url=https%3A%2F%2Fen.wikipedia.org%2Fwiki%2FEscherichia_coli&usg=AFQjCNFT_fJrAAEMjuPEQYfRdOqM5ifGCw&sig2=a3UiOKgWSQVOdmhHygmBEg)  0157: H7 ATCC 43890 |
| 119 | × | × | ✓ | × | ✓ | ✓ | ✓ | [*E. coli*](https://www.google.co.kr/url?sa=t&rct=j&q=&esrc=s&source=web&cd=2&cad=rja&uact=8&ved=0ahUKEwi9reTfoYvPAhWGVZQKHa4iAs4QFggiMAE&url=https%3A%2F%2Fen.wikipedia.org%2Fwiki%2FEscherichia_coli&usg=AFQjCNFT_fJrAAEMjuPEQYfRdOqM5ifGCw&sig2=a3UiOKgWSQVOdmhHygmBEg)  0157: H7 ATCC 43890 |
| 120 | ✓ | ✓ | × | ✓ | ✓ | ✓ | ✓ | [*L. monocytogenes*](https://www.google.co.kr/url?sa=t&rct=j&q=&esrc=s&source=web&cd=1&cad=rja&uact=8&ved=0ahUKEwjr1om9oovPAhWCoJQKHQXfAHcQFggiMAA&url=https%3A%2F%2Fen.wikipedia.org%2Fwiki%2FListeria_monocytogenes&usg=AFQjCNGclwOGCJ_vA7pTFhCwISnMtBS3kg&sig2=U-NJNe3xcw6TWk9ePvp35g) ATCC 19115 |
| 121 | ✓ | ✓ | × | ✓ | ✓ | ✓ | ✓ | [*L. monocytogenes*](https://www.google.co.kr/url?sa=t&rct=j&q=&esrc=s&source=web&cd=1&cad=rja&uact=8&ved=0ahUKEwjr1om9oovPAhWCoJQKHQXfAHcQFggiMAA&url=https%3A%2F%2Fen.wikipedia.org%2Fwiki%2FListeria_monocytogenes&usg=AFQjCNGclwOGCJ_vA7pTFhCwISnMtBS3kg&sig2=U-NJNe3xcw6TWk9ePvp35g) ATCC 19115 |
| 122 | ✓ | ✓ | × | ✓ | ✓ | ✓ | ✓ | [*L. monocytogenes*](https://www.google.co.kr/url?sa=t&rct=j&q=&esrc=s&source=web&cd=1&cad=rja&uact=8&ved=0ahUKEwjr1om9oovPAhWCoJQKHQXfAHcQFggiMAA&url=https%3A%2F%2Fen.wikipedia.org%2Fwiki%2FListeria_monocytogenes&usg=AFQjCNGclwOGCJ_vA7pTFhCwISnMtBS3kg&sig2=U-NJNe3xcw6TWk9ePvp35g) ATCC 19115 |
| 123 | ✓ | × | × | × | × | × | × | [*S.* *aureus*](https://www.google.co.kr/search?biw=1920&bih=934&q=staphylococcus+aureus&spell=1&sa=X&ved=0ahUKEwjTp_GboovPAhXEm5QKHYQJBgkQvwUIFygA) KCCM 40050 |
| 124 | × | × | ✓ | × | ✓ | ✓ | ✓ | Dead cell |
| 125 | × | ✓ | × | ✓ | × | ✓ | ✓ | [*B. subtilis*](https://www.google.co.kr/url?sa=t&rct=j&q=&esrc=s&source=web&cd=1&cad=rja&uact=8&ved=0ahUKEwihsbGnoovPAhVBGJQKHfQwDV8QFggdMAA&url=https%3A%2F%2Fen.wikipedia.org%2Fwiki%2FBacillus_subtilis&usg=AFQjCNGFijWm9LtsdZ-Da63OlFj9u3JOdg&sig2=w2ehAcPeNMdxfmDxDgXppA&bvm=bv.132653024,d.dGo)  ATCC 14579 |
| 126 | × | × | ✓ | × | ✓ | ✓ | ✓ | Dead cell |
